# Local genetic variation of inflammatory bowel disease in Basque population and its effect in risk prediction

**DOI:** 10.1038/s41598-022-07401-2

**Published:** 2022-03-01

**Authors:** Koldo Garcia-Etxebarria, Olga Merino, Adrián Gaite-Reguero, Pedro M. Rodrigues, Amaia Herrarte, Ane Etxart, David Ellinghaus, Horacio Alonso-Galan, Andre Franke, Urko M. Marigorta, Luis Bujanda, Mauro D’Amato

**Affiliations:** 1 Biodonostia, Gastrointestinal Genetics Group, 20014 San Sebastián, Spain; 2 Centro de Investigación Biomédica en Red de Enfermedades Hepáticas y Digestivas (CIBERehd), Barcelona, Spain; 3 Gastroenterology Department, Hospital Universitario Cruces, Barakaldo, Spain; 4 Integrative Genomics Lab, Center for Cooperative Research in Biosciences (CIC bioGUNE), Basque Research and Technology Alliance (BRTA), Bizkaia Technology Park, Derio, Basque Country Spain; 5 Biodonostia, Liver Diseases Group, 20014 San Sebastián, Spain; 6 IKERBASQUE, Basque Foundation for Sciences, Bilbao, Spain; 7 Biodonostia, Gastrointestinal Disease Group, Universidad del País Vasco (UPV/EHU), 20014 San Sebastián, Spain; 8 Institute of Clinical Molecular Biology, Christian-Albrechts-University of Kiel, Kiel, Germany; 9 Gastroenterology Department, Hospital Universitario Donostia, 20014 San Sebastián, Spain; 10 Gastrointestinal Genetics Lab, CIC bioGUNE, Basque Research and Technology Alliance, 48160 Derio, Spain

**Keywords:** Genome-wide association studies, Inflammatory bowel disease, Medical genomics

## Abstract

Inflammatory bowel disease (IBD) is characterised by chronic inflammation of the gastrointestinal tract. Although its aetiology remains unknown, environmental and genetic factors are involved in its development. Regarding genetics, more than 200 *loci* have been associated with IBD but the transferability of those signals to the Basque population living in Northern Spain, a population with distinctive genetic background, remains unknown. We have analysed 5,411,568 SNPs in 498 IBD cases and 935 controls from the Basque population. We found 33 suggestive *loci* (p < 5 × 10^−6^) in IBD and its subtypes, namely Crohn’s Disease (CD) and Ulcerative Colitis (UC), detecting a genome-wide significant *locus* located in HLA region in patients with UC. Those *loci* contain previously associated genes with IBD (*IL23R*, *JAK2* or HLA genes) and new genes that could be involved in its development (*AGT*, *BZW2* or *FSTL1*). The overall genetic correlation between European populations and Basque population was high in IBD and CD, while in UC was lower. Finally, the use of genetic risk scores based on previous GWAS findings reached area under the curves > 0.68. In conclusion, we report on the genetic architecture of IBD in the Basque population, and explore the performance of European-descent genetic risk scores in this population.

## Introduction

Inflammatory bowel disease (IBD) comprises different entities characterized by the presence of chronic inflammatory and relapsing damages in the gastrointestinal tract, especially in the small intestine and in the colon. Its most important subtypes are Crohn’s Disease (CD) and Ulcerative Colitis (UC). The former can be located in any part of the gastrointestinal tract and it is characterized by transmural inflammation; while the latter is usually located in the colon and it is confined to the mucosa. The most common symptoms developed by IBD patients include diarrhoea, anaemia, abdominal pain and weight loss^1^.

Although its aetiology remains unknown, epidemiological and genetic data suggest that IBD is triggered by environmental factors in genetically-predisposed individuals. As consequence of those factors, there is an excessive inflammatory response that causes the symptomatology. Among the environmental factors, infections and tobacco consumption have been proposed, but ample uncertainty remains in this area^1^. The genetic component of IBD has been analysed using genome-wide association studies (GWAS). More than 200 risk *loc*i have been identified in European ancestry and patients from other ethnicities. In addition, the majority of those risk *loci* are common for CD and UC, with similar effects; and among other signals, some independent signals in the human leukocyte antigen (HLA) region have been previously described^2–4^.

However, those risk *loci* explain only a minor proportion of the observed heritability of IBD and, as it happens in other complex diseases, the prevalence of the IBD and associated genetic risk variants associated with IBD vary across populations^5,6^. For example, *NOD2* gene has been associated with CD in some European populations, but the evidence for association in a Scottish population was lower^6^. Known biological sources of heterogeneity between populations include differences due to variation in allele frequency (for example, in *NOD2* gene), effect size (for example, *TNFSF15* and *ATG16L1* genes) or the combinations of both (for example, *IL23R* and *IRGM* genes)^3^.

The availability of genetic information permits to develop Polygenic Risk Scores (PRS) for IBD. The promise of PRS is the stratification of patients according to their genetic variants and the risk of developing a complex disease. Based on the carriership of risk alleles, an individual can be identified as more prone to develop the disease, with the entailed potential to translate the genetic knowledge into clinical practice^7^. However, a general theme across complex diseases is that the performance of the application of PRS is dependent on the population, even if they are from the same ethnicity^8–10^.

The Basque population shows some genetic differences compared to the rest of European populations, probably due to their isolation and the effect of genetic drift. As consequence of that particular genetic history, the Basque population has retained more genetic makeup related to populations that lived in Europe in the Neolithic^11^ or Iron Age^12^, with less impact from latter migrations associated to the Steppe pastoralism. For example, the Basque population shows a slightly different frequency of the haplotypes of HLA region^13^, as aforementioned, a region associated to IBD^2^. Of note, according to the Basque Statistic Institute (https://en.eustat.eus), between 2016 and 2019, in the Basque Autonomous Community (Northern Spain) there were 2804 hospitalizations involving 27,789 days of hospital stays due to IBD.

Our aim with this study is two-fold. First, to characterize for the first time the genetic architecture of IBD in the Basque region, a population that presents genetic particularities within the general European genetic background that has been profusely studied in GWAS for IBD. Secondly, in order to explore the transferability of genetic risk estimators across population, we study the performance of European-based polygenic risk scores in the Basque population, therefore, to infer the utility of the genetic information for IBD in the clinical practice among different populations.

## Results

In the present study we have analysed 498 IBD cases, of which 284 were CD cases and 208 UC cases, and 935 healthy controls (Table 1). We found that the patients with IBD were older than the controls (41.46 years ± 11.85 vs 51.42 years ± 13.97, respectively; t-test p = 9.11 × 10^−38^). In addition, the proportion of females was higher in patients with IBD (48.59%) when compared with controls (32.83%). Regarding the clinic features of the disease, the majority of CD cases had ileal (46.5%) or ileocolonic location (40.8%); and for UC, more than half of the cases had left-sided extension (50.5%, Table 1).

**Table 1:** Demographics and features of the Basque cohort analysed in the present study.

	Inflammatory bowel disease	Crohn’s disease	Ulcerative colitis	Controls
N	498	284	208	935
Male	256 (51.4%)	141 (49.6%)	113 (54.3%)	628 (67.2%)
Female	242 (48.6%)	143 (51.4%)	95 (45.7%)	307 (32.8%)
Age (SE)	51.4 (13.9)			41.46 (11.9)
**Disease location**				
Ileal	–	132 (46.5%)	–	–
Colorectal	–	30 (10.6%)	–	–
Ileocolonic	–	116 (40.8%)	–	–
Upper GI	–	6 (2.1%)	–	–
**Disease extent**				
Proctitis	–	–	24 (11.5%)	–
Left-sided	–	–	105 (50.5%)	–
Extensive	–	–	72 (34.6%)	–
**Disease behaviour**				
Inflammatory	–	191(67.2%)	–	–
Stricturing	–	61 (21.5%)	–	–
Penetring	–	64 (22.5%)	–	–

**Figure 1:**
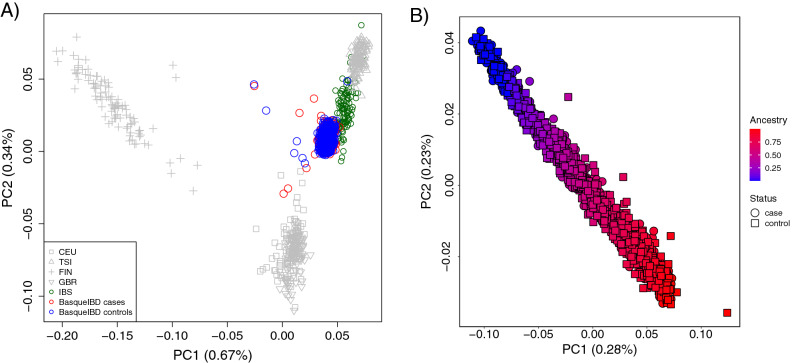
Genetic background of the Basque cohort analysed in the present study. (**A**) Relationship of the Basque cohort within 1000 genomes project European populations, according to Principal Component Analysis. (**B**) Principal Component Analysis of the Basque cohort, coloured by their ancestry according to Admixture analysis. Graphics were depicted using R language 4.0.5 (https://www.r-project.org) and ggplot2 3.3.5 (https://ggplot2.tidyverse.org).

We first established the genetic background of our cohort and its placement in the context of European populations (Fig. 1A). The genetic background of our cohort overlapped with Iberian population of 1000 Genomes Project, although some of the analysed individuals distanced from the core of the Iberian population (Fig. 1A). In more detail, we analysed the first two principal components of the genetic distance between individuals and we did not detect any particular clustering (Fig. 1B). Due to the particular genetic history of the Basque population, we analysed the admixture of our cohort, where two ancestral groups had the lowest cross-validation results. The first two principal component reflected the ancestry component of each individual, placing them into a general continuity of the mixture of the two inferred ancestral populations (Fig. 1B), and we used that information as covariate in the GWAS analysis.

### Genome-Wide association study

In the GWAS we evaluated 5,411,568 SNPs to find differences in allele frequency between patients with IBD (cases) and healthy controls. We found that 41 SNPs had suggestive significance (p < 5 × 10^−6^) when all IBD cases were analysed, 25 SNPs when only CD cases were analysed and 49 SNPs when only UC cases were analysed. Those SNPs were located in 12, 14 and 12 suggestive *loci*, respectively (Table 2), for a total of 33 unique *loci* study-wide. From those signals, we found one genome-wide significant signal in UC (Table 2), in HLA region (rs41291790, p = 2.9 × 10^−8^, OR = 5.3). That association, as well another 3 *loci*, were previously associated with IBD or its subtypes (Table 2), according to the PheWAS analysis. Among the genes mapped in the suggestive *loci*, we found genes previously linked to IBD and its subtypes (such as *IL23R*, *JAK2* or genes located in HLA region), as well as genes not previously associated to IBD or its subtypes, including among others, *AGT*, *BZW2* or *FSTL1* genes, located on *loci* where the lead SNP had an OR of 2.0 (95% of confidence interval of 1.5–2.7), 3.2 (2.1–5.1) and 1.5 (1.3–1.8), respectively (Table 2). On the whole, regardless of their significance, the direction of the effect of those suggestive signals was concordant in CD and UC in all the lead SNPs except for one (Table 2).

**Table 2:** Basque IBD GWAS association results and annotation, suggestive *loci*.

Lead SNP	Position	EA	OA	EAF	IBD (498/935)		CD (284/935)		UC (208/935)		Nearest gene	Previously known
					p	OR	p	OR	UC	UC		
rs871822	chr1:3312914	G	T	0.266	**0.002**	0.8	0.739	1.0	**3.8E−06**	0.5	PRDM16	
rs10082259	chr1:4495821	C	T	0.832	**1.1E−04**	1.5	**6.2E−07**	1.9	0.294	1.2	RNF220	
rs17436816	chr1:6204161	A	G	0.112	**0.001**	1.6	**3.3E−06**	2.0	0.739	1.1	CHD5	
rs77894461	chr1:19385772	T	C	0.051	**3.1E−06**	2.2	**0.003**	1.9	**7.6E−06**	2.7	UBR4	
rs6702829	chr1:29766241	C	T	0.232	**2.1E−06**	0.6	**2.2E−04**	0.6	**0.001**	0.6		
rs6660226	chr1:67744601	A	G	0.453	**1.9E−06**	0.7	**8.9E−06**	0.6	**0.005**	0.7	IL23R	IBD, CD, UC
rs1710775	chr1:106081424	C	A	0.670	**1.1E−04**	1.4	**2.9E−06**	1.7	0.172	1.2		
rs12566217	chr1:230792242	G	A	0.144	**1.8E−04**	1.5	0.103	1.3	**1.5E−06**	2.0	COG2	
rs12185578	chr2:17609239	A	T	0.282	**4.2E−06**	1.5	**1.3E−04**	1.5	**4.4E−04**	1.5	RAD51AP2	
rs148746268	chr2:25605572	G	A	0.022	**1.6E−04**	2.7	**2.9E−06**	3.8	0.563	1.3	DTNB	
rs72949953	chr2:214026384	A	G	0.039	**3.1E−05**	2.3	**0.025**	1.8	**1.2E−06**	3.2	IKZF2	
rs11129387	chr3:30221063	A	T	0.364	**0.001**	0.7	**4.4E−06**	0.5	0.639	0.9	RBMS3	
rs62266031	chr3:116539468	G	C	0.366	**2.9E−06**	0.6	**7.0E−05**	0.6	**0.001**	0.6		
rs2030413	chr3:120082782	A	G	0.464	**3.5E−07**	1.5	**1.7E−06**	1.6	**0.008**	1.3	LRRC58	
rs9307388	chr4:114075688	T	A	0.027	**1.2E−05**	2.9	**6.6E−07**	3.8	**0.045**	2.0	ANK2	
rs143431075	chr4:173051881	A	G	0.025	**3.6E−04**	2.5	**3.6E−06**	3.6	0.623	1.2	GALNTL6	
rs3121685	chr5:65662133	C	T	0.532	**3.6E−06**	1.5	**9.9E−05**	1.5	**1.2E−04**	1.6		
rs2052483	chr5:102948185	T	C	0.214	**3.2E−06**	1.6	**4.5E−06**	1.8	**0.016**	1.4	NUDT12	
rs10515625	chr5:148647553	C	T	0.105	**3.8E−06**	0.5	**0.001**	0.5	4.4E**−**04	0.5	AFAP1L1	
rs3910312	chr6:30008746	C	A	0.255	**5.0E−05**	1.4	0.087	1.2	**6.7E−07**	1.8	ZNRD1ASP	IBD, UC
rs41291790	chr6:31572664	A	G	0.022	**2.4E−05**	3.1	0.160	1.7	**2.9E−08**	5.3	AIF1	IBD, CD, UC
rs6946352	chr7:16706549	G	T	0.045	**3.5E−06**	2.4	**0.007**	1.9	**2.7E−07**	3.2	BZW2	
rs10952655	chr7:146391520	A	G	0.782	**0.001**	1.4	**2.3E−06**	1.8	0.951	1.0	CNTNAP2	
rs4370571	chr8:138304595	A	G	0.582	**0.002**	0.8	0.573	0.9	**4.3E−06**	0.6	LOC101927915	
rs10119004	chr9:5071049	G	A	0.543	**7.8E−06**	0.7	**2.0E−06**	0.6	0.053	0.8	JAK2	IBD, CD, UC
rs75486977	chr10:63579821	T	C	0.015	**0.001**	2.7	0.781	1.1	**4.8E−06**	4.9	CABCOCO1	
rs17231595	chr10:68724763	T	G	0.017	**0.001**	2.8	0.244	1.6	**4.6E−06**	4.8	CTNNA3	
rs1826333	chr11:27850631	C	A	0.721	**9.9E−06**	1.4	**3.9E−06**	1.6	**0.031**	1.3	BDNF	
rs2806899	chr13:46483515	A	G	0.622	**1.6E−06**	0.7	**0.001**	0.7	**1.2E−04**	0.6	ZC3H13	
rs177206	chr14:78538806	C	T	0.466	**0.001**	1.3	**2.6E−06**	1.6	0.808	1.0	NRXN3	
rs1998136	chr14:92280675	A	G	0.188	**5.7E−07**	0.5	**4.8E−05**	0.5	**0.001**	0.5	TC2N	
rs72755010	chr15:64304844	C	T	0.042	**0.002**	1.8	0.636	1.1	**3.0E−06**	2.8	DAPK2	
rs11648328	chr16:4618931	C	T	0.265	**3.5E−05**	1.5	**3.4E−07**	1.8	0.246	1.2	C16orf96	

Below the analysis, between brackets, the number of cases and controls analysed.

*EA* effect allele; *OA* other allele; *EAF* effect allele frequency; *IBD* results from the analysis of all IBD patients; *CD* results from the analysis of only Crohn’s Disease patients; *UC* results from the analysis of only Ulcerative Colitis patients; *p* p-value of the effect allele, in bold nominally significant results; *OR* odds-ratio of the effect allele; *Nearest gene* nearest gene within 100 kb from lead SNP (if any).

We observed further association in some of those signals with location or extent of disease (Table 3). In the case of CD, 5 *loci* were more significantly associated with ileal CD than in ileocolonic CD, for example, rs1826333 (ileal CD p = 1.7E−07, ileocolonic CD p = 0.084); while 7 *loci* were more significant in ileocolonic CD than in ileal CD, for example, rs11129387 (ileal CD p = 0.034, ileocolonic CD p = 7.7E−06). In the case of UC, 8 *loci* were more significantly associated with left-sided extension than in extensive extension, for example, rs871822 (left-sided UC p = 2.7E−05, extensive UC p = 0.006); while 8 *loci* were more significantly associated with extensive extension than in left-sided extension, for example, rs17231595 (left-sided UC p = 0.020, extensive UC p = 4.3E−07).

**Table 3:** Basque IBD GWAS association results in each subtype*,* suggestive *loci*.

Lead SNP	Ileal CD (132/935)		Ileocolinic CD (116/935)		Left-sided UC (105/935)		Extensive UC (72/935)	
	p	OR	p	OR	p	OR	p	OR
rs871822	0.600	0.9	0.727	1.1	**2.7E−05**	0.4	**0.006**	0.5
rs10082259	**0.001**	1.7	**3.1E−05**	2.0	0.055	1.4	0.971	1.0
rs17436816	**5.8E−05**	2.2	**9.2E−05**	2.2	0.581	1.2	0.735	0.9
rs77894461	**0.001**	2.3	0.273	1.4	**0.001**	2.5	**0.003**	2.7
rs6702829	0.140	0.8	**0.004**	0.6	**0.005**	0.6	0.373	0.8
rs6660226	**0.003**	0.7	**4.2E−04**	0.6	**0.047**	0.7	**0.032**	0.7
rs1710775	**0.001**	1.7	**0.002**	1.7	0.754	1.1	0.701	1.1
rs12566217	0.644	1.1	0.084	1.4	**0.001**	2.0	**4.0E−04**	2.2
rs12185578	**0.016**	1.4	**0.001**	1.6	**0.027**	1.4	**0.024**	1.5
rs148746268	**7.3E−05**	4.2	**0.002**	3.3	0.870	1.1	0.414	0.4
rs72949953	0.211	1.6	**0.009**	2.3	**5.1E−05**	3.4	**4.2E−04**	3.4
rs11129387	**0.034**	0.7	**7.7E−06**	0.4	0.447	0.9	0.454	0.8
rs62266031	**0.013**	0.7	**0.008**	0.6	**0.009**	0.6	0.081	0.7
rs2030413	**3.0E−04**	1.6	**0.001**	1.6	0.132	1.2	**0.013**	1.6
rs9307388	**6.6E−05**	3.9	**1.5E−05**	4.4	**0.012**	2.9	0.629	1.4
rs143431075	**1.1E−04**	3.9	**3.9E−04**	3.6	0.291	1.7	0.373	0.4
rs3121685	**0.011**	1.4	**0.002**	1.6	**2.8E−04**	1.8	0.101	1.3
rs2052483	**0.003**	1.6	**4.9E−04**	1.8	0.055	1.4	0.174	1.4
rs10515625	**0.001**	0.4	0.171	0.7	**0.007**	0.4	0.079	0.6
rs3910312	0.158	1.2	0.152	1.3	**0.001**	1.7	**0.001**	1.8
rs41291790	0.671	1.3	0.281	1.7	**5.6E−06**	5.4	**4.8E−05**	5.6
rs6946352	0.075	1.8	**0.015**	2.1	**0.032**	2.0	**7.9E−07**	4.7
rs10952655	**0.001**	1.8	**0.004**	1.6	0.753	0.9	0.577	1.1
rs4370571	**0.008**	0.7	**0.042**	1.3	**0.003**	0.6	**8.2E−05**	0.5
rs10119004	**0.003**	0.7	**0.001**	0.6	0.364	0.9	**0.006**	0.6
rs75486977	0.486	1.5	0.396	0.4	**1.6E−05**	5.7	**0.002**	4.7
rs17231595	0.166	2.1	0.392	0.4	**0.020**	3.1	**4.3E−07**	8.4
rs1826333	**1.7E−07**	2.0	0.084	1.3	**0.008**	1.5	0.772	1.1
rs2806899	**0.001**	0.6	0.763	1.0	0.073	0.8	**0.002**	0.5
rs177206	**0.001**	1.6	**4.4E−05**	1.8	0.942	1.0	0.874	1.0
rs1998136	**0.005**	0.5	**0.006**	0.5	**0.009**	0.5	0.070	0.6
rs72755010	0.806	1.1	0.459	1.3	**4.1E−04**	2.7	**8.6E−05**	3.4
rs11648328	**1.3E−05**	1.9	**0.001**	1.8	0.169	1.3	0.940	1.0

Below the subtype, between brackets, the number of cases and controls analysed. p, p-value of the effect allele, in bold nominally significant results; OR, odds-ratio of the effect allele.

We further characterized the results through gene-set enrichment analyses and alternative methods for gene mapping. While the physically genes located in *loci* in IBD and CD do not show any significant enrichment, in UC, due to the markers located in HLA region, those genes belonged mainly to immunity related function, such as innate immune response, interferon gamma mediated signalling or antigen processing and presentation (Supplementary Table S1). However, when we used alternative gene mapping strategies, namely Depict and S-PrediXcan methods, we did not obtain any significant result after multiple test correction.

Moreover, we examined the significant *loci* from the results of International IBD Genetic Consortium (IIBDGC) in our cohort. On the whole, we observed few lead SNPs located in those *loci* involved in IBD or its subtypes were nominally significant in Basque cohort (Supplementary Table S2). In total, we found 25 of those *loci* nominally significant in IBD, 27 in CD and 23 in UC; and the direction of the effect was consistent between IIBDGC results and our cohort in 21, 23, and 18 *loci*, respectively (Supplementary Table S2).

Considering the size and the allele frequencies in our cohort, we calculated the statistical power to replicate nominally (p < 0.05) the signals detected in IIBDGC. We concluded that our power to replicate those signals at p < 0.05 was up to 36, 35 and 24 for IBD, CD and UC, respectively. From those signals we detected a nominal p-value in 24, 25 and 21 *loci*, respectively. Therefore, the effective replicability rate of IIBDGC signals in the Basque cohort was 67% for IBD, 71.4% for CD, and 87.5% for UC; and we detected a nominal p-value in one signal in IBD, 2 signals in CD and 2 signals in UC that, theoretically, we have not enough power.

Finally, we selected some of the most relevant genes well-known to be associated to IBD, namely, *IL23R*, *ATG26L1*, *IRGM*, *TNFSF15*, *LRRK2* and *NOD2* to study in detail the evidence of association in our cohort (Supplementary Table S3). In the case of *IL23R* and *NOD2* genes, we showed that the significance of some SNPs located in those genes was higher when only CD cases were analysed that in all IBD cases; namely rs11209023 in *IL23R* and rs5743292 SNPs in *NOD2*. The significance of those SNPs in each location of CD (ileal or ileocolonic) was similar for *IL23R*; while in *NOD2* some SNPs were more significant in ileocolonic CD than colonic CD, such us, for example rs5743292 (Supplementary Table S3). When we analysed the SNPs located in *LRRK2* gene, there were SNPs whose significance was higher when all IBD cases were analysed than analysing each subtype separately (rs4767970); and their significance was higher in ileal CD than in ileocolonic CD, and in left UC than in pancolitis UC (rs4767970). In the rest of the analysed genes in detail, such as, *ATG26L1*, *IRGM* or *TNFSF15*, we did not find any relevant signal (Supplementary Table S3).

### Heritability and genetic correlations

The estimated heritability was calculated using LDSC: the heritability of IBD in our cohort was h^2^ = 0.579 ± 0.338 and, in the case of the subtypes, the estimate was particularly larger for CD (h^2^ = 0.773 ± 0.411) than for UC (h^2^ = 0.464 ± 0.362). Therefore, Z score of the heritability was 1.71 for IBD, 1.88 for CD and 1.28 for UC, all values below the significance threshold (Z score > 1.96 for p = 0.05).

**Figure 2:**
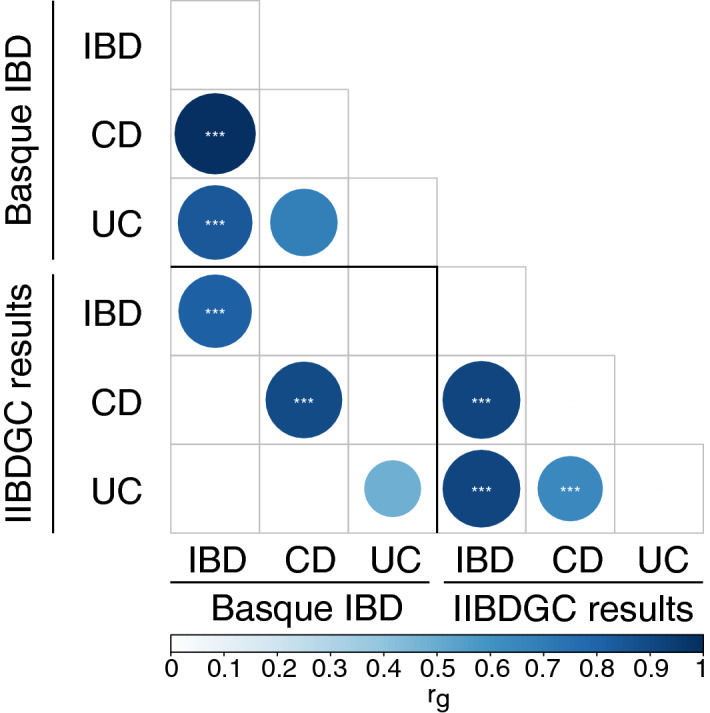
Genetic regression of the results of the present study and their counterparts from IIBDGC, for IBD and its subtypes. Circle size and colour depict regression coefficients. Inside the circle the significance of the regression coefficient, ***p < 0.001, **p < 0.01, *p < 0.05; otherwise, not significant.

Regarding the genetic correlation analysis carried out using LDSC program, we found that IBD and CD GWAS findings from the Basque cohort were significantly correlated with their counterparts from IIBDGC, with a significant regression score: 0.817 ± 0.235 (p = 0.0005) and 0.892 ± 0.235 (p = 0.0001) respectively; while the genetic overlap was not significant in UC (Fig. 2). Furthermore, in the Basque cohort there was significant correlation between IBD and CD (p = 2.14 × 10^−29^); and IBD and UC (p = 0.0001); but not between CD and UC; while in the results from IIBDGC IBD and its subtypes were genetically correlated between them (Fig. 2).

In addition, we carried out a genetic correlation analysis with the traits available in CTG-VL and LDHub tools. The top hits were IBD and its subtypes, but after False Discovery Rate correction, we did not find any significant genetic correlation with those traits.

### HLA association analysis

In the analysis of HLA imputation using HIBAG, we found 19 HLA alleles associated with IBD, CD or UC (Table 4). Eight of those alleles were significant when all IBD patients were analysed; 10 when only CD patients were analysed; and 9 when only patients with UC were analysed (Table 4). The most significant haplotype was HLA_A_0201 in UC (p = 1.21 × 10^−5^, OR = 1.99), a signal previously known in UC (Table 4). Among the haplotypes, we found that 7 haplotypes were not previously associated with IBD or its subtypes (Table 4).

**Table 4:** HLA imputation association results in the Basque IBD cohort, significant alleles.

HLA allele	MAF	IBD (498/935)		CD (284/935)		UC (208/935)		Previously known
		p	OR	p	OR	p	OR	
HLA_A_3301	0.016	**0.032**	0.4	0.087	0.4	0.163	0.5	
HLA_A_2902	0.092	0.264	1.2	**0.049**	1.4	0.552	0.9	UC
HLA_A_0201	0.262	**0.002**	1.4	0.336	1.1	**1.2E-05**	2.0	UC
HLA_A_3002	0.037	**0.027**	0.6	0.060	0.6	0.165	0.6	
HLA_A_6801	0.018	**0.007**	0.3	**0.018**	0.2	0.166	0.5	
HLA_C_0102	0.035	**0.033**	0.6	**0.024**	0.5	0.411	0.8	
HLA_C_1601	0.087	0.111	1.3	**0.040**	1.4	0.834	1.0	UC
HLA_C_0702	0.125	0.470	1.1	0.605	0.9	**0.049**	1.4	UC
HLA_B_0702	0.122	0.311	1.1	0.668	0.9	**0.015**	1.5	UC
HLA_DRB1_1101	0.063	0.360	1.2	0.722	0.9	**0.044**	1.5	UC
HLA_DRB1_0301	0.107	**0.010**	0.7	**0.016**	0.6	0.175	0.8	CD, UC
HLA_DRB1_0701	0.193	0.953	1.0	**0.039**	1.3	**0.014**	0.6	CD, UC
HLA_DQA1_0104	0.024	0.545	1.2	0.394	0.7	**0.043**	1.9	
HLA_DQA1_0201	0.193	0.890	1.0	**0.038**	1.3	**0.020**	0.7	CD, UC
HLA_DQA1_0501	0.107	**0.016**	0.7	**0.026**	0.7	0.191	0.8	
HLA_DQB1_0503	0.024	0.545	1.2	0.394	0.7	**0.043**	1.9	
HLA_DQB1_0201	0.106	**0.013**	0.7	**0.018**	0.6	0.191	0.8	CD, UC
HLA_DQB1_0202	0.162	0.464	0.9	0.193	1.2	**0.003**	0.6	UC
HLA_DPB1_1101	0.062	0.230	1.2	**0.035**	1.5	0.735	0.9	UC

Below the subtype, between brackets, the number of cases and controls analysed.

*MAF* minor allele frequency; *IBD* results from the analysis of all IBD patients; *CD* results from the analysis of only Crohn’s Disease patients; *UC* results from the analysis of only Ulcerative Colitis patients; *p* p-value of the allele, in bold nominally significant results; *OR* odds-ratio of the allele.

### Application of polygenic risk score

**Figure 3:**
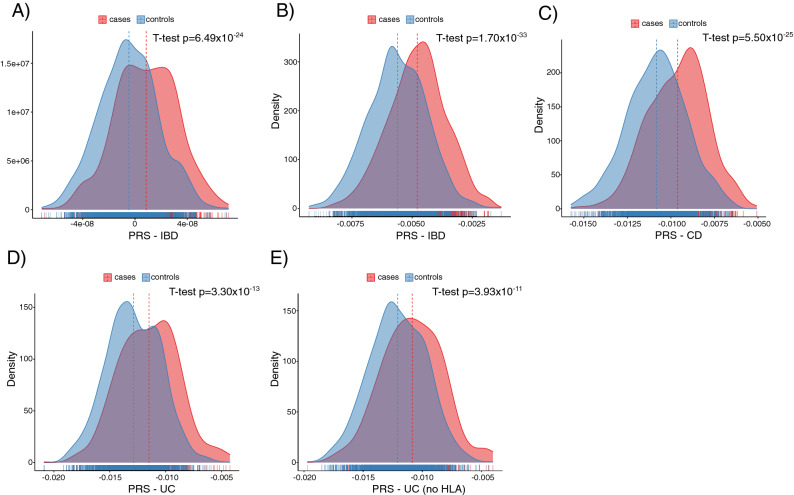
Polygenic risk score (PRS) analysis of IBD and its subtypes. T-test p, p-value of the T-test comparing the PRS scores of cases and controls. (**A**) PRS calculated for all Inflammatory Bowel Disease samples using the PRS derived in Khera et al.^7^. (**B**) Optimal PRS calculated for all Inflammatory Bowel Disease samples using IIBDGC results as model. (**C**) Optimal PRS calculated only for Crohn’s Disease samples using IIBDGC results as model. (**D**) Optimal PRS calculated only for Ulcerative Colitis samples using IIBDGC results as model. (**E**) Optimal PRS calculated only for Ulcerative Colitis samples, excluding HLA region, using IIBDGC results as model.

Firstly, we applied to our Basque cohort a set of publicly available polygenic risk scores (PRS) previously derived from GWAS analyses of UK Biobank as described in Khera et al.^7^ (Fig. 3A) and available through PGS catalog. In total, we could use in our cohort the weights of 5,913,246 SNPs from that PRS model. The Area Under the Curve (AUC) value was 0.69 (Confidence Interval of 95% 0.66–0.72) and the difference of the mean PRS score between IBD cases and controls was significant (t-test p of 6.49 × 10^−24^).

Then, in order to derive Basque-specific PRS, we computed polygenic risk scores in the Basque cohort by using summary statistics from the IIBDGC GWAS results, using PRSice-2 (Fig. 3B–E). The best PRS models included 809 SNPs markers for IBD (at a p-value threshold of 0.0002), 733 SNPs for CD (p-value threshold of 0.0002) and 303 SNPs for UC (p-value threshold of 5 × 10^−05^). With the limitation that we used these PRS in the same population used to generate them (lack of independent replication cohort), the accuracy of a prediction model was higher in IBD and CD, with AUC values of 0.72 (CI of 95% 0.69–0.74) and 0.73 (CI of 95% 0.69–0.76), respectively, than in UC (AUC of 0.68, CI of 95% 0.63–0.72). Accordingly, the difference of the mean PRS score between cases and controls (again from the same cohort) was more significant in IBD and CD (t-test p of 1.70 × 10^−33^ and 5.50 × 10^−25^, respectively, Fig. 3B and C) than in UC (p of 3.30 × 10^−13^, Fig. 3D). Since UC showed a bimodal distribution both in cases and controls, we removed the HLA region from the PRS calculation (Fig. 3E), using 295 SNPs (p-value threshold of 5 × 10^−05^) in the best model. This led to a distribution resembling normality, but the AUC was lower (0.66, CI of 95% 0.62–0.70) and the comparison of the average scores was less significant (t-test p of 3.93 × 10^−11^).

## Discussion

In the present study we have analysed for the first time the genetic architecture of inflammatory bowel disease (IBD) and its main subtypes, Crohn’s Disease (CD) and Ulcerative colitis (UC), in a cohort from the Basque region. Although the small sample size of our study hampers the discovery of significant signals, our results provide clues about the transferability of genetic findings in European populations not studied to date, especially in those with particular genetic history as the current Basques.

It has been established that the Basque population has been less affected by the admixture processes that shaped the modern European genetic pool, maintaining more ancestry fractions from the Neolithic^11^ and the Iron Age^12^. Indeed, likely composed of “modern Basques”, our cohort reflected such an admixed nature, with the two first PC possibly reflecting the effect of the mentioned historical processes. Thus, we incorporated the correction of PC to avoid spurious results in the GWAS analysis, due to the effect of a possible subtle stratification, as it has been previously used successfully in a more complex admixed populations^14^.

The genetic architecture of IBD and its subtypes have been established in different cohorts and populations, mainly from European ancestry cohorts^3,15^. Compared with those studies, the number of patients of each subtype and the location and behaviour of the disease in our cohort was slightly different. For example, in our cohort the inflammatory behaviour of CD represented 67% of the CD cases while in Cleynen et al. was 50%. In addition, we have shown genetic differences between the different localization or extension of the disease, both in suggestive *loci* and in SNPs located in different genes. Those differences could be an effect of the sampling, the results of environmental effects^16^ or a reflect of local genetic differences and, therefore, those could affect our results and our comparison with what is established in IBD and its subtypes.

We have found one genome-wide significant result: rs41291790 in the HLA region in UC, that was previously associated to IBD and its subtypes. The rest of signals are suggestive, some of them associated previously to IBD or its subtypes; and the overlap of known associated *loci*^3^ and their significance in our cohort was scarce. However, considering the expected replicability in our cohort, we captured 67–87% of the expected signals, suggesting slight differences that could be affected by different genetic architecture or environmental effects; and that is important to study different populations to capture all the heterogeneity. In addition, when the whole genetic background is considered, we showed that IBD and CD correlated better with what is known from IIBDGC results^3^ (r_g_ > 0.8) whereas, in the Basque population, the overlap of UC with European populations was lower. In fact, in IIBDGC results, CD and UC seem to share partially the genetic architecture^3^, while in our cohort the genetic overlap was not significant. The same can be concluded from heritability analyses: although they were not significant, the heritability of CD was higher than UC in our cohort. In addition, on the whole, the direction of the effects of genetic variants in Basque cohort were concordant between subtypes, and with the ones from IIBDGC. In the case of *loci* that were not previously associated with IBD further replication analyses are needed to stablish their relevance. Moreover, and considering all the limitations of our cohort, we were able to detect differences in the effects of suggestive *loci* depending on the location or extension of the disease, as it has been previously described^15^. Genetic heterogeneity between populations have been previously described in IBD^5,6^, and, since the genetic background our population is slightly different from the rest of European populations, it is to be expected that there are slightly genetic differences, as we have found. Therefore, although the sample size of our cohort and its statistical power could be a limitation to discover new strong signals, even more so considering the possible influence from differences in the linkage disequilibrium in the Basques, we were able to detect the main features of the genetic architecture of IBD.

As mentioned, the strongest signals in UC in Basque population are located in HLA region, the previously mentioned rs41291790, and rs3910312, which are associated with IBD, according to the PheWAS analysis. In addition, the strongest HLA allelic association in the Basque cohort (HLA_A_0201) had higher OR than IIBDGC results (1.99 in Basque cohort, 1.14 in IIBDGC results^2^); and we have detected new HLA alleles that has not been associated to IBD or its subtypes. It is well established that HLA is a genomic region associated with UC and its behaviour^2,4^ and, therefore, our results are consistent with the involvement of HLA region in UC. In addition, the frequency of the haplotypes of HLA region is slightly different in the Basques^13^ or Northern Spain^17^ from other European populations; and it has been established that the risk haplotypes of HLA in rheumatoid arthritis in Basques were different to other populations^18^; as well as for multiple sclerosis^19^. Thus, the results we obtained in the HLA region in UC are consistent with the observation in other complex diseases that the involvement of HLA alleles is slightly different in the Basque population.

A complementary way to infer the strengths and limits of our results is to inspect individual genes. *NOD2* is a gene that is associated with CD, especially with ileum affectation^15^, it is known to vary in association patterns across populations, even for near groups^6^, and it has been pointed out as the source of the risk to CD in European and non-European admixed populations^20,21^. Our results, although not genome-wide significant, are consistent with those observations: we found almost suggestive significance of *NOD2* in CD and in some SNPs more significant results in ileal CD. *LRRK2* gene have been associated with IBD^3,22^, specially with CD^3,22^, and another chronic inflammatory diseases^23^. In our results we see that is significant in IBD, and there are not relevant differences between subtypes. *LRRK2* gene is also well known to be a risk gene in Parkinson Disease, and one of the known mutations that confers more risk in that disease has its origin in the Basque population, while that mutation is scarcer in other populations^24^. Thus, although more refined work is need to understand the haplotype effects in this genomic region, this might suggest that *LRRK2* presents differences in effects in the Basque population, since that gene is an example of a gene that reflects the distinctive genetic background of the Basque population^24^.

Moreover, as mentioned before, we detect some suggestive *loci* that require further validation in a Basque cohort. Among the genes located on those *loci*, we found *AGT* gene, a gene involved in the genetic risk of thromboembolic events in IBD^25^; in the prognosis of colorectal cancer^26^, a cancer whose risk is increased in CD^27^; and it has been proposed that *AGT* is an important regulator of apoptosis in the intestinal epithelial cells^28^. In addition, other genes located in those suggestive *loci* are *BZW2* gene, a possible oncogene that could be a driver gene in colorectal cancer^29^; *DAPK2* gene, a gene involved in the progression of colorectal cancer^30^; and *FSTL1* gene, a gene involved in proinflammatory response in inflammatory diseases^31^. Due to the biological mechanism where those genes are involved, although suggestive, those genes seem good candidate genes for follow-up analyses to understand the development and prognosis of IBD, at least in the Basque population. Therefore, the role of the mentioned genes in the development of IBD should be established in future studies, at least in Basque cohort.

Considering the genetic correlations and that some genes showed consistent involvement in IBD and CD compared with other European populations, it seems that the genetic architecture of IBD and CD in the Basque population is more similar to other European population, while the genetic architecture of UC was slightly different.

The use of the PRS derived from UK Biobank^7^ in IBD showed a slightly better performance than in that work (AUC of 0.69 in our cohort, 0.63 in UK Biobank^7^). When a Basque-specific PRS model was derived using IIBDGC GWAS results, the performance was slightly better in IBD (AUC value of 0.72), although with the important limitation that the same population was used both to derive the PRS and to test them for their discriminative potential (possibly generating inflated results). In the case of CD, the most optimal model had an AUC of 0.73, which is lower than other studies^32,33^. In one study^32^, first IIBDGC data from 4906 CD cases and 11,494 controls was used to derived the PRS using different methods, such as, mixed linear models, elastic net regularization or Bayesian methods, to get the best predictive model. Then the best model was applied in 2204 CD cases and 997 controls from Australia and New Zeeland and the highest AUC was 0.78^32^. In other study^33^, 112 SNPs were tested to build the most optimal model for PRS in Slovenian population, where 202 CD cases and 236 controls were analysed; and the best AUC was 0.78 using 33 SNPs^33^. In the case of UC, the performance of the most optimal model (AUC = 0.68) in our cohort was not as good as IBD and CD. The lower performance of PRS in UC than in CD was previously observed^32^: using 5788 UC cases and 16,194 controls from IIBDGC data to construct the best model and then applying it in 1193 UC cases and 997 controls from Australia and New Zeeland, the best AUC was 0.70^32^. Therefore, the most optimal model used in the present work should be analysed in an independent Basque cohort to validate its applicability. In addition, considering the good performance of IIBDGC panel in Basque and other cohorts, it seems that application of PRS in IBD and CD should be based in data generated from multiple populations and, in this way, be useful in the clinical practice in different populations. As mentioned, the case of UC seems to be slightly different. Although we removed the HLA region from the PRS calculation to avoid the slightly different allelic frequencies in the Basque population^13^, the performance of PRS did not improve. Therefore, that translation of genetic results of UC to clinical practice seems more complicated, as it has been previously described in other complex diseases in the use of PRS in close populations^8,9^. In conclusion, it seems that the performance of PRS reflected the differences in the genetic architectures of IBD and its subtypes.

On the whole, we explored genetic features of IBD and its subtypes in a small Basque cohort for the first time. We detected signals mostly compatible and overlapping with those previously described in large multicentre cohorts of European descent, further suggesting the potential transferability of GWAS findings across European populations. Some of the association signals detected here in the Basques, may correspond to *bona fide* risk loci and variants specific to this population, which warrants further investigation in much larger samples from the same area.

## Methods

### Samples

IBD cases were diagnosed using standard criteria; and the samples used in this study were obtained in the standard clinical practice, after informed consenting, in Hospital Universitario Donostia (San Sebastian, Spain) and Hospital Universitario de Cruces (Barakaldo, Spain). The samples of non-IBD controls were obtained through the Basque Biobank. In total 549 cases were recruited and 987 controls were used. All participants provided written informed consent.

The present study was approved by the Local Ethics Committee (Comité de Ética de la Investigación con medicamentos de Euskadi, code: PI + CES-BIOEF 2017-10).

### Genotyping and imputation

DNA samples from the individuals included in this study were genotyped using Illumina Global Screening Array on Illumina iScan high-throughput screening system in the Institute of Clinical Molecular Biology (Kiel, Germany). To call the alleles from raw intensities the GenCall algorithm available in Illumina GenomeStudio 2.0 (https://www.illumina.com/techniques/microarrays/array-data-analysis-experimental-design/genomestudio.html) software was used.

Genotyped data was filtered removing samples and markers using the following procedure: exclusion of samples with ≥ 15% missing rates; exclusion of markers with non-called alleles; exclusion of markers with missing call rates > 0.05; exclusion of samples with ≥ 5% missing rates; exclusion of related samples (PI-HAT > 0.1875); exclusion of samples whose genotyped sex could not be determined; exclusion of samples with high heterozygosity rate (more than three times SD from the mean); only autosomal SNPs were kept; removal of markers with Hardy–Weinberg equilibrium p < 1 × 10^−5^; removal of markers whose p of difference in missingness between cases and control was < 1 × 10^−5^; and removal of samples which were outliers, identified using principal component analysis (deviation of more than six times interquartile range).

Imputation of missing genotyped was done using the Sanger Imputation service. The reference panel used was the release 1.1 of Haplotype Reference Consortium and the pipeline used was EAGLE2 + PBWT^34–36^. Once imputed, markers with INFO score < 0.80, MAF < 0.01 and non-biallelic markers were removed.

After genotyping, quality control and imputation, 5,411,568 SNPs from 1433 individuals (498 cases and 935 controls) were kept.

### Genetic analyses

#### Admixture analysis

Genotyped SNPs were pruned using Plink^37^ and SNPs from regions with high linkage disequilibrium were removed. Considering the particular genetic history of our cohort, a population admixture analysis was carried out using Admixture^38^, setting K between 1 and 10, and using the results with lowest cross-validation value. The analysis was carried out using the samples of our cohort.

#### Genome-wide association studies

GWAS analyses were performed using logistic regression implemented in Plink^37^, adjusting by sex and first four principal components. The analyses were performed with all IBD cases, as well as only CD cases and only UC cases separated.

In addition, ileal CD (N = 132), ileocolinic CD (N = 116), left-sided UC (N = 105) and extensive UC (N = 72) were separately analysed using logistic regression implemented in Plink^37^, adjusting by sex and first 4 principal components.

#### *Loci* definition and gene-mapping

Risk *loci* from the analysed phenotypes were defined as non- overlapping genomic regions extending a linkage disequilibrium window (r^2^ = 0.4) from the association signals with p < 5.0 × 10^−6^. Annotation of GWAS results, including genes mapping to the identified risk loci, was performed with functional mapping and annotation (FUMA) of GWAS^39^.

### Power analysis

195 independent genome-wide significant loci from IIBDGC results were selected^3^. To study the statistical power to replicate the IBDGC signals in the Basque IBD GWAS, a power analysis was carried out using the R package “genpwr”^40^. The power calculation was performed for all IBD subtypes (i.e., IBD, Cd and UC) separately.

Replicating SNPs were defined as SNPs with nominally significant p-values (p < 0.05) in our study. Expected number of replicating SNPs can be estimated as the sum of the power to attain nominal replication of every IIBDGC SNP. The ratio between observed and expected number of SNPs permits to calculate the effective replicability rate.

### PheWAS analysis

Lead SNPs from each suggestive *locus* was inspected using Phenoscanner V2^41,42^. Traits associated to the Lead SNP or with SNPs in LD with the Lead SNP (R^2^ ≥ 0.8) were retrieved; and traits with genome-wide significant p-value (p < 5 × 10^−8^) were kept.

### Gene-set enrichment analyses

To test for over-representation of biological functions based on gene annotations (gene set enrichment analysis), we screened the Molecular Signature Database (MsigDB) using the list of FUMA mapped genes against all genes in hypergeometric enrichment tests. Gene sets with an adjusted p < 0.05 (false discovery rate correction according to Benjamini–Hochberg) were considered significant evidence of enrichment.

Depict^43^, as it is available in CTG-VL (https://vl.genoma.io), was used to find the causal genes at associated loci and to perform an gene-set enrichment and tissue enrichment analyses. In that analysis SNPs with p < 1 × 10^−5^ were used.

S-PrediXcan, an extension of PrediXcan for summary data, was used to map genes through expression data of relevant tissues^44^, as it is available in CTG-VL. The expression data used was based on GTEx^45^ and the tissues inspected were terminal ileum, colon transverse and colon sigmoid. Genes with p < 2.5E−7 were considered significant. In addition, gene set enrichment analyses with those genes were performed using FUMA.

### Heritability and genetic correlation

To study the heritability and genetic correlation of the results of this study and the results from IIBDGC ldsc program^46^ was used, as it is available in CTG-VL. Results from all IBD cases, only CD cases and only UC cases association analyses of the present study were compared with their counterparts available from IIBDGC. In addition, we analysed the genetic correlations of IBD, CD and UC association analyses with the traits available in CTG-VL and LDHub^47^.

### HLA association analysis

HLA types were imputed from genotyped data using HIBAG package^48^ available in R language^49^. In the imputation European panel was used as model.

The association analysis was carried out with HIBAG using logistic regression and testing dominant model, adjusting by sex and first four principal components.

The analyses were performed with all IBD cases, as well as only CD cases and only UC cases separated.

### Polygenic risk score

Firstly, Polygenic risk score (PRS) was calculated using the weights calculated by by Khera et al.^7^ and retrieved from PGS catalog^50^. Those weights were applied in the Basque cohort using Plink^37^.

Secondly, PRS were calculated using PRSice software^51^. As base summary statistics the results from IIBDGC was used; additive model was tested; and the analysis was adjusted by sex and first four principal components. The analyses were performed with all IBD cases, as well as only CD cases and only UC cases separated. The performance of the PRS was measured comparing the PRS score distribution of cases and controls using a T-test using R language^49^; and calculating the area under de curve using pROC package of R language. The 95% of confidence interval of the area under the curve was calculated using that package and DeLong method.

Graphics were depicted using R language^49^, and ggplot2 3.3.5^52^ and corrplot 0.87 (https://github.com/taiyun/corrplot) packages.

All methods were performed in accordance with relevant guidelines and regulations including the Declarations of Helsinki.

## Supplementary Information

Supplementary Information.

## Data Availability

The genotypes generated in this work will be incorporated to the International IBD Genetic Consortium. The summary statistics will be available in GWAS Catalog, under the accession GCST90020070–GCST90020072. All the results have been included as supplementary information.
