# Supplementary material for: Local genetic variation of inflammatory bowel disease in Basque population and its effect in risk prediction
Source: Sci Rep. 2022 Mar 1;12:3386. doi: 10.1038/s41598-022-07401-2 (PMC8888637; doi:10.1038/s41598-022-07401-2)
Supplement: Supplementary file 1 — Supplementary Information. [file 41598_2022_7401_MOESM1_ESM.pdf]

Local genetic variation of inflammatory bowel disease in Basque population and its effect in risk prediction

Koldo Garcia-Etxebarria[1,2,*], Olga Merino[3], Adrián Gaite-Reguero[4], Pedro M. Rodrigues[2,5,6], Amaia Herrarte[7], Ane Etxart[7], David Ellinghaus[8], Horacio Alonso-Galan[7,9], Andre Franke[8], Urko M. Marigorta[4,6], Luis Bujanda[2,7], Mauro D'Amato[1,6,10]

[1] Biodonostia, Gastrointestinal Genetics Group, 20014 San Sebastián, Spain
[2] Centro de Investigación Biomédica en Red de Enfermedades Hepáticas y Digestivas (CIBERehd)
[3] Gastroenterology Department, Hospital Universitario Cruces, Barakaldo, Spain
[4] Integrative Genomics Lab, Center for Cooperative Research in Biosciences (CIC bioGUNE), Basque Research and Technology Alliance (BRTA), Bizkaia Technology Park, Derio, Basque Country, Spain
[5] Biodonostia, Liver Diseases Group. 20014 San Sebastián, Spain
[6] IKERBASQUE, Basque Foundation for Sciences, Bilbao, Spain
[7] Biodonostia, Gastrointestinal Disease Group. Universidad del País Vasco (UPV/EHU). 20014 San Sebastián, Spain
[8] Institute of Clinical Molecular Biology, Christian-Albrechts- University of Kiel, Kiel, Germany
[9] Gastroenterology Department, Hospital Universitario Donostia. 20014 San Sebastián, Spain.
[10] Gastrointestinal Genetics Lab, CIC bioGUNE, Basque Research and Technology Alliance, 48160 Derio, Spain

	**Page**
**Supplementary Table S1**: Gene-Set Enrichment Analysis of mapped genes in suggestive *loci* of UC	2
**Supplementary Table S2**: GWAS association results of Basque IBD cohort in the significant *loci* from IIBDGC results.	3
**Supplementary Table S3**: Genome-Wide Association Studies of IBD, its subtypes and analysed location and extension for selected genes.	8

Supplementary Table S1: Gene-Set Enrichment Analysis of mapped genes in suggestive *loci* of UC

Category	GeneSet	Adjusted-P
KEGG	Allograft rejection	0.000125
KEGG	Graft versus host disease	0.000125
KEGG	Type i diabetes mellitus	0.0001269
KEGG	Autoimmune thyroid disease	0.0002133
GO_mf	Peptide antigen binding	0.0002508
GO_bp	Interferon gamma mediated signaling pathway	0.0002781
KEGG	Viral myocarditis	0.0005177
KEGG	Complement and coagulation cascades	0.0005177
GO_bp	Antigen processing and presentation of endogenous peptide antigen via mhc class i via er pathway	0.0006504
GO_bp	Innate immune response	0.0006504
GO_bp	Positive regulation of apoptotic cell clearance	0.0006504
GO_bp	Antigen processing and presentation of peptide antigen via mhc class ib	0.0006504
GO_bp	Antigen processing and presentation of exogenous peptide antigen via mhc class i tap independent	0.0006504
GO_bp	Positive regulation of immune response	0.0006504
GO_bp	Regulation of apoptotic cell clearance	0.0007906
KEGG	Antigen processing and presentation	0.0007975
GO_bp	Positive regulation of immune system process	0.0013887
GO_bp	Regulation of immune effector process	0.0013887
GO_bp	Antigen processing and presentation of endogenous peptide antigen	0.0019454
GO_bp	Response to interferon gamma	0.0024242
GO_bp	Defense response	0.0024242
GO_bp	Antigen processing and presentation via mhc class ib	0.0024242
GO_bp	Regulation of immune response	0.0028644
KEGG	Cell adhesion molecules cams	0.0042314
KEGG	Systemic lupus erythematosus	0.0042314
GO_bp	Negative regulation of viral entry into host cell	0.0044894
GO_mf	Antigen binding	0.0047744
GO_bp	Antigen processing and presentation of endogenous antigen	0.0049633
GO_bp	Complement activation	0.0059925
GO_bp	Regulation of humoral immune response	0.0059925
GO_bp	Lymphocyte mediated immunity	0.0059925
GO_mf	Complement binding	0.0062947
GO_bp	Adaptive immune response based on somatic recombination of immune receptors built from immunoglobulin superfamily domains	0.0065435
GO_bp	Positive regulation of t cell mediated cytotoxicity	0.0076778
GO_bp	Regulation of viral entry into host cell	0.0125148
GO_bp	Activation of immune response	0.0125148
GO_bp	Regulation of t cell mediated cytotoxicity	0.0144127
GO_bp	Negative regulation of viral process	0.0166684
GO_bp	Movement in environment of other organism involved in symbiotic interaction	0.0170575
GO_bp	Regulation of immune system process	0.0170575
GO_bp	Interaction with host	0.0238219
GO_bp	B cell mediated immunity	0.029712
GO_bp	Apoptotic cell clearance	0.029712
GO_bp	Natural killer cell cytokine production	0.029712
GO_bp	Regulation of symbiosis encompassing mutualism through parasitism	0.029712
GO_bp	Positive regulation of t cell mediated immunity	0.029712
GO_mf	Beta 2 microglobulin binding	0.0298153
GO_bp	Humoral immune response mediated by circulating immunoglobulin	0.0328797
GO_bp	T cell mediated cytotoxicity	0.0340445
GO_bp	Adaptive immune response	0.0430361
GO_bp	Entry into other organism involved in symbiotic interaction	0.0431917

Supplementary Table S2: GWAS association results of Basque IBD cohort in the significant *loci* from IIBDGC results.

			IBD				CD				UC			
			IIBDGC		BasqueIBD		IIBDGC		BasqueIBD		IIBDGC		BasqueIBD	
**IIBDGC - SNP**	**CHR:POS**	**BasqueIBD SNP**	**P**	**OR**	**P**	**OR**	**P**	**OR**	**P**	**OR**	**P**	**OR**	**P**	**OR**
rs12103	1:1247494	rs11590283 (R2=0.956)	1.1E-05	1.1	0.118	0.9	0.003	1.1	0.172	0.8	5.0E-05	1.1	0.343	0.9
rs10910092	1:2501516	rs10910092	4.2E-07	1.1	0.137	1.1	0.320	1.0	0.191	1.1	3.3E-09	1.1	0.536	1.1
rs72634258	1:8150638	rs72634258	4.6E-10	1.2	0.180	1.2	0.005	1.1	0.246	1.2	5.7E-09	1.2	0.508	1.1
rs6426833	1:20171860	rs6426833	1.4E-16	1.2	0.142	1.1	0.890	1.0	0.166	1.1	4.9E-31	1.3	0.301	1.1
rs34920465	1:22700351	rs34920465	8.2E-10	1.1	0.050	1.2	0.092	1.1	0.275	1.2	2.9E-11	1.2	0.047	1.4
rs631106	1:62901807	rs631106	0.014	1.0	0.417	1.1	1.0E-05	1.1	0.380	1.1	0.721	1.0	0.516	1.1
rs7547569	1:67731368	rs7547569	7.9E-61	2.0	0.001	1.7	1.4E-52	2.7	0.002	2.0	1.3E-24	1.7	0.033	1.7
rs10889881	1:71023684	rs10889881	0.682	1.0	0.327	1.1	0.222	1.0	0.870	1.0	0.353	1.0	0.163	1.2
rs17391694	1:78623626	rs17391694	0.017	0.9	0.438	0.9	4.0E-04	0.9	0.694	0.9	0.954	1.0	0.408	0.9
rs34856868	1:92554283	rs34856868	0.271	0.9	0.065	0.7	0.490	0.9	0.030	0.5	0.420	0.9	0.610	0.9
rs11583043	1:101466054	rs11583043	0.004	1.1	0.606	1.0	0.524	1.0	0.807	1.0	2.5E-04	1.1	0.655	0.9
rs2476601	1:114377568	rs2476601	0.084	1.0	0.594	0.9	2.5E-07	0.8	0.413	0.8	0.049	1.1	0.840	1.0
rs2641348	1:120437884	rs2641348	0.007	1.1	0.908	1.0	2.1E-04	1.2	0.139	1.3	0.305	1.0	0.138	0.8
rs4845604	1:151801680	rs11801866 (R2=0.589)	1.6E-05	0.9	0.843	1.0	0.049	0.9	0.607	1.1	2.2E-05	0.9	0.339	0.8
rs490608	1:155682870	rs490608	1.2E-04	1.1	0.212	1.1	6.4E-07	1.1	0.675	1.0	0.152	1.0	0.095	1.2
rs2297559	1:160854526	rs2297559	4.3E-06	1.1	0.524	1.1	4.2E-04	1.1	0.851	1.0	6.8E-05	1.1	0.420	1.1
rs10800309	1:161472158	rs7522794 (R2=1.0)	1.2E-15	1.2	0.017	1.2	1.0E-04	1.1	0.016	1.3	6.8E-15	1.2	0.424	1.1
rs6025	1:169519049	rs77977611 (R2=0.2497)	0.001	0.8	0.513	1.2	0.051	0.9	0.814	1.1	0.012	0.8	0.443	1.3
rs6425143	1:172844352	rs6425143	3.7E-04	0.9	0.989	1.0	4.9E-14	0.8	0.857	1.0	0.372	1.0	0.741	1.0
rs10798069	1:186875459	rs10798069	0.104	1.0	0.081	1.2	0.001	0.9	0.108	1.2	0.992	1.0	0.234	1.1
rs2488397	1:197701279	rs2488397	4.4E-04	1.1	0.116	1.2	1.0E-04	1.1	0.274	1.1	0.092	1.0	0.328	1.1
rs76181804	1:198601705	rs76181804	0.068	1.0	0.141	0.8	0.001	0.9	0.706	0.9	0.996	1.0	0.027	0.7
rs2816958	1:200101920	rs2816958	0.001	0.9	0.823	1.0	0.694	1.0	0.546	1.1	2.2E-06	0.8	0.207	0.8
rs35730213	1:200874229	rs35730213	6.9E-15	0.9	0.014	0.8	3.7E-06	0.9	0.010	0.7	8.8E-12	0.8	0.324	0.9
rs3024493	1:206943968	rs3024505 (R2=0.9858)	8.5E-22	1.2	0.174	1.2	5.2E-09	1.2	0.436	1.1	1.1E-17	1.3	0.234	1.2
rs13407913	2:25097644	rs13407913	0.001	0.9	0.017	0.8	0.003	0.9	0.023	0.8	0.017	1.0	0.188	0.9
rs780094	2:27741237	rs780094	2.2E-04	1.1	0.235	1.1	2.9E-04	1.1	0.437	1.1	0.004	1.1	0.190	1.2
rs2279990	2:28636740	rs2279990	8.8E-05	1.1	0.056	1.2	0.001	1.1	0.389	1.1	0.019	1.1	0.015	1.3
rs78487399	2:43809347	rs78487399	4.9E-09	1.2	0.247	0.9	1.0E-09	1.3	0.119	0.8	0.002	1.1	0.942	1.0
rs7608910	2:61204856	rs7608910	7.0E-16	0.9	0.090	0.9	9.8E-08	0.9	0.006	0.8	1.8E-12	0.9	0.961	1.0
rs11679753	2:62552321	rs10865331 (R2=0.9834)	0.998	1.0	0.696	1.0	0.005	1.1	0.592	0.9	0.045	1.0	0.899	1.0
rs6740462	2:65667272	rs6740462	0.002	1.1	0.407	0.9	0.010	1.1	0.040	0.8	0.025	1.1	0.357	1.1
rs1420098	2:102984279	rs1420098	3.6E-07	1.1	0.111	1.1	2.1E-05	1.1	0.287	1.1	2.1E-04	1.1	0.159	1.2
rs11681525	2:145492382	rs11681525	1.2E-04	1.1	0.428	1.2	3.5E-04	1.2	0.632	1.1	0.024	1.1	0.313	1.3
rs4664304	2:160794008	rs4664304	0.025	1.0	0.872	1.0	0.606	1.0	0.751	1.0	0.021	1.1	0.859	1.0
rs1990760	2:163124051	rs1990760	0.002	0.9	0.795	1.0	0.110	1.0	0.348	0.9	0.001	0.9	0.471	1.1
rs1517352	2:191931464	rs1517352	9.6E-06	0.9	0.872	1.0	3.4E-04	0.9	0.245	0.9	1.8E-04	0.9	0.375	1.1
rs1595825	2:198875464	rs1595825	0.060	1.0	0.830	1.0	0.383	1.0	0.552	0.9	4.4E-04	0.9	0.803	1.0
rs17229285	2:199523122	rs10931828 (R2=0.9568)	0.013	1.0	0.177	1.1	0.855	1.0	0.150	1.2	6.4E-05	0.9	0.684	1.0
rs3116494	2:204592021	rs3116494	4.0E-04	0.9	0.461	1.1	0.189	1.0	0.401	1.1	4.8E-04	0.9	0.846	1.0
rs11677953	2:219121663	rs11677953	1.1E-08	1.1	0.583	1.0	0.006	1.1	0.649	1.0	2.8E-08	1.1	0.215	1.1

rs7556897	2:228660112	rs4973341 (R2=1.0)	0.001	1.1	0.133	1.1	0.116	1.0	0.541	1.1	0.001	1.1	0.121	1.2
rs9989735	2:231115454	rs9989835 (R2=1.0)	6.9E-05	1.1	2.0E-04	1.5	1.2E-08	1.2	0.002	1.5	0.181	1.0	0.004	1.5
rs6738490	2:234161583	rs6738490	1.1E-17	0.9	0.041	0.8	1.1E-37	0.7	0.010	0.8	0.083	1.0	0.607	0.9
rs4676410	2:241563739	rs4676410	8.8E-12	1.2	0.058	1.2	0.006	1.1	0.111	1.2	2.5E-13	1.2	0.181	1.2
rs35320439	2:242737341	rs35320439	0.213	1.0	0.152	1.1	0.260	1.0	0.210	1.1	0.032	1.1	0.347	1.1
rs11708026	3:18741802	rs11708026	3.3E-06	0.9	0.383	1.1	6.9E-07	0.9	0.616	1.1	0.002	0.9	0.397	1.2
rs113010081	3:46457412	rs113010081	0.003	0.9	0.903	1.0	0.709	1.0	0.482	1.2	4.9E-06	0.9	0.427	0.8
rs9836291	3:49697459	rs9836291	6.0E-23	1.2	0.719	1.0	2.5E-13	1.2	1.000	1.0	4.7E-15	1.2	0.339	0.9
rs9847710	3:53062661	rs9847710	0.109	1.0	0.975	1.0	0.014	1.1	0.880	1.0	2.5E-06	0.9	0.999	1.0
rs616597	3:101569726	rs616597	0.021	1.0	0.856	1.0	0.008	0.9	0.484	1.1	0.387	1.0	0.314	0.9
rs724016	3:141105570	rs724016	0.086	1.0	0.712	1.0	1.7E-04	0.9	0.574	0.9	0.416	1.0	0.902	1.0
rs2073505	4:3444503	rs75501914 (R2=0.3626)	1.6E-05	1.1	0.312	1.2	1.1E-04	1.2	0.564	1.1	0.009	1.1	0.228	1.3
rs4692386	4:26132361	rs11732941 (R2=0.1076)	5.6E-05	0.9	0.096	0.8	1.5E-05	0.9	0.170	0.8	0.045	1.0	0.277	0.8
rs6856616	4:38325036	rs6856616	0.001	0.9	0.350	1.2	0.001	0.9	0.286	1.2	0.032	0.9	0.626	1.1
rs7438704	4:48363245	rs7438704	0.250	1.0	0.619	1.0	0.001	0.9	0.156	0.9	0.522	1.0	0.311	1.1
rs2457996	4:74856535	rs352009 (R2=1.0)	0.009	1.1	0.991	1.0	0.964	1.0	0.766	1.0	1.7E-04	1.1	0.902	1.0
rs3774937	4:103434253	rs3774937	0.141	1.0	0.987	1.0	0.302	1.0	0.148	1.2	0.002	0.9	0.077	0.8
rs10010325	4:106106353	rs2903385 (R2=0.996)	5.0E-06	0.9	0.377	0.9	1.6E-04	0.9	0.424	0.9	7.5E-05	0.9	0.619	0.9
rs7657746	4:123161619	rs7657746	1.2E-04	1.1	0.277	1.1	0.001	1.1	0.165	1.2	0.006	1.1	0.693	1.1
rs11739663	5:594083	rs11739663	4.7E-04	1.1	0.106	1.2	0.611	1.0	0.229	1.2	9.3E-07	1.1	0.306	1.1
rs3776414	5:10689562	rs3776414	3.7E-05	0.9	0.320	0.9	0.001	0.9	0.213	0.9	4.2E-04	0.9	0.953	1.0
rs395157	5:38867732	rs395157	1.5E-05	1.1	0.056	1.2	4.8E-04	1.1	0.016	1.3	0.001	1.1	0.551	1.1
rs6880778	5:40399096	rs6880778	2.1E-27	0.8	0.071	0.9	6.7E-34	0.7	0.003	0.7	7.0E-07	0.9	0.649	1.1
rs71624119	5:55440730	rs71624119	0.007	0.9	0.072	0.8	0.002	0.9	0.062	0.8	0.262	1.0	0.379	0.9
rs4703855	5:71693899	rs4703855	2.2E-04	0.9	0.825	1.0	0.001	0.9	0.965	1.0	0.020	0.9	0.819	1.0
rs34804116	5:72539850	rs34804116	0.001	0.9	0.108	0.9	2.7E-05	0.9	0.173	0.9	0.378	1.0	0.338	0.9
rs1363907	5:96252803	rs1363907	1.9E-07	1.1	0.005	1.3	1.5E-11	1.2	0.017	1.3	0.080	1.0	0.045	1.3
rs55722650	5:131607300	rs55722650	9.9E-15	1.1	0.176	1.1	6.0E-18	1.2	0.193	1.1	3.6E-05	1.1	0.496	1.1
rs56399423	5:131672657	rs56399423	9.4E-17	0.9	0.455	0.9	3.1E-20	0.8	0.319	0.9	5.5E-06	0.9	0.952	1.0
rs254562	5:134441457	rs254562	3.2E-09	0.9	0.269	0.9	0.035	1.0	0.714	1.0	9.7E-09	0.9	0.184	0.9
rs449454	5:141533062	rs449454	8.9E-06	0.9	0.030	0.8	7.5E-08	0.9	0.081	0.8	0.025	1.0	0.119	0.8
rs17800987	5:150323428	rs17800987	3.7E-11	0.8	0.960	1.0	4.7E-15	0.7	0.518	0.9	2.0E-04	0.9	0.342	1.2
rs56167332	5:158827769	rs755374 (R2=0.9604)	3.3E-17	1.2	0.643	1.0	2.3E-11	1.2	0.620	1.1	5.3E-11	1.2	0.844	1.0
rs564349	5:172324978	rs564349	4.1E-04	0.9	0.676	1.0	0.003	0.9	0.954	1.0	0.002	0.9	0.354	0.9
rs56163845	5:173373948	rs56163845	0.040	1.0	0.015	1.3	0.001	1.1	0.085	1.2	0.851	1.0	0.019	1.4
rs4976646	5:176788570	rs4976646	3.8E-04	0.9	0.568	1.1	0.085	1.0	0.773	1.0	4.3E-04	0.9	0.604	1.1
rs7773324	6:382559	rs7773997 (R2=0.972)	0.197	1.0	0.360	0.9	0.013	1.1	0.573	1.1	0.681	1.0	0.020	0.8
rs13204048	6:3420406	rs9328169 (R2=0.9434)	0.006	1.1	0.361	1.1	8.3E-05	1.1	0.471	1.1	0.400	1.0	0.374	1.1
rs17119	6:14719496	rs17119	5.4E-09	1.1	0.559	1.1	1.4E-06	1.2	0.921	1.0	5.2E-06	1.1	0.319	1.1
rs2328546	6:20657345	rs2328546	8.6E-08	0.9	0.511	0.9	1.7E-07	0.9	0.938	1.0	3.5E-04	0.9	0.348	0.9
rs12663356	6:21430728	rs12663356	3.6E-04	0.9	0.861	1.0	1.4E-05	0.9	0.679	1.0	0.309	1.0	0.878	1.0
rs9264942	6:31274380	rs9264942	4.3E-08	0.9	0.747	1.0	1.9E-13	0.8	0.807	1.0	0.055	1.0	0.335	1.1
rs9271100	6:32576478	rs9271109 (R2=1.0)	2.7E-20	1.2	0.861	1.0	0.446	1.0	0.025	0.8	9.4E-35	1.4	0.039	1.3
rs1847472	6:90973159	rs1847472	5.8E-07	0.9	0.368	1.1	3.9E-06	0.9	0.813	1.0	0.002	0.9	0.077	1.2
rs4946717	6:106474749	rs4349854 (R2=0.9763)	2.9E-05	0.9	0.921	1.0	0.001	0.9	0.981	1.0	0.001	0.9	0.692	1.0
rs2179070	6:111885752	rs145220678 (R2=1.0)	2.4E-08	0.8	0.018	1.5	0.001	0.9	0.891	1.0	6.3E-07	0.8	0.001	2.0

rs2503322	6:127457260	rs2503322	0.022	1.0	0.992	1.0	2.3E-05	0.9	0.830	1.0	0.999	1.0	0.862	1.0
rs9491891	6:128277151	rs9491891	0.113	1.0	0.615	1.1	2.6E-04	0.9	0.443	1.1	0.468	1.0	0.970	1.0
rs6933404	6:137959235	rs6933404	4.8E-08	0.9	0.335	0.9	0.202	1.0	0.842	1.0	3.7E-11	0.8	0.127	0.8
rs12199775	6:143898894	rs12199775	8.9E-05	1.1	0.838	1.0	0.006	1.1	0.900	1.0	0.010	1.1	0.708	1.1
rs7758080	6:149577079	rs7758080	5.0E-04	0.9	0.028	0.8	0.005	0.9	0.158	0.9	0.061	1.0	0.023	0.8
rs212388	6:159490436	rs629326 (R2=0.9835)	0.012	1.0	0.103	0.9	6.4E-07	0.9	0.211	0.9	0.855	1.0	0.214	0.9
rs444210	6:167390242	rs444210	7.4E-11	0.9	0.004	0.8	1.0E-12	0.8	0.034	0.8	0.001	0.9	0.027	0.8
rs1182188	7:2869985	rs1182188	8.2E-05	1.1	0.611	1.0	0.700	1.0	0.712	1.0	1.4E-07	1.1	0.217	1.2
rs1077773	7:17442679	rs9969271 (R2=0.7593)	0.013	1.0	0.356	1.1	0.820	1.0	0.777	1.0	0.001	1.1	0.164	1.2
rs10486483	7:26892440	rs10486483	0.048	1.0	0.001	1.4	0.001	1.1	0.001	1.5	0.354	1.0	0.075	1.3
rs4722672	7:27231762	rs4722672	3.4E-05	0.9	0.186	1.1	0.055	0.9	0.138	1.2	3.9E-05	0.9	0.655	1.1
rs10276381	7:28190121	rs6945537 (R2=1.0)	0.075	1.0	0.713	1.0	0.030	1.1	0.208	0.8	0.748	1.0	0.460	1.1
rs1456896	7:50304461	rs1456896	1.4E-07	1.1	0.066	1.2	2.9E-08	1.1	0.109	1.2	0.013	1.1	0.247	1.2
rs2395022	7:98750379	rs2395022	1.5E-05	1.2	0.127	1.4	0.003	1.2	0.136	1.4	3.4E-04	1.2	0.275	1.3
rs314313	7:100423365	rs314314 (R2=0.9908)	6.0E-05	0.9	0.026	0.8	0.001	0.9	0.456	0.9	0.004	0.9	0.005	0.7
rs6466198	7:107480126	rs6466198	1.3E-12	1.1	0.074	1.2	0.009	1.1	0.194	1.1	2.0E-13	1.2	0.098	1.2
rs38911	7:116895163	rs38911	4.4E-04	0.9	0.659	1.0	0.060	1.0	0.990	1.0	2.1E-04	0.9	0.295	1.1
rs4728142	7:128573967	rs4728142	0.003	1.1	0.220	1.1	0.389	1.0	0.485	1.1	2.8E-07	1.1	0.149	1.2
rs2538470	7:148220448	rs2538470	0.002	1.1	0.079	0.9	4.7E-04	1.1	0.151	0.9	0.077	1.0	0.252	0.9
rs17057051	8:27227554	rs17057051	0.001	1.1	0.335	1.1	0.002	1.1	0.292	1.1	0.038	1.0	0.599	1.1
rs7011507	8:49129242	rs7814627 (R2=1.0)	1.7E-04	0.9	0.848	1.0	0.046	0.9	0.844	1.0	0.001	0.9	0.763	1.1
rs7015630	8:90875918	rs2338883 (R2=0.995)	0.003	1.1	0.685	1.0	1.5E-05	1.1	0.568	0.9	0.964	1.0	0.255	1.2
rs10956252	8:126536137	rs55867617 (R2=1.0)	5.0E-09	0.9	0.473	0.9	6.5E-12	0.8	0.128	0.9	0.007	0.9	0.555	1.1
rs6651252	8:129567181	rs6651252	0.010	1.1	0.762	1.0	3.4E-06	1.2	0.174	1.3	0.607	1.0	0.259	0.8
rs13277237	8:130604563	rs13277237	1.0E-06	0.9	0.031	0.8	0.002	0.9	0.030	0.8	3.4E-05	0.9	0.145	0.8
rs10758669	9:4981602	rs36051895 (R2=0.7904)	1.1E-20	0.8	9.8E-05	1.4	4.5E-11	0.9	6.8E-05	1.6	4.1E-14	0.8	0.039	1.3
rs4743820	9:93928416	rs4743820	3.5E-06	1.1	0.038	1.2	0.002	1.1	0.254	1.1	3.6E-05	1.1	0.041	1.3
rs7848647	9:117569046	rs7848647	5.6E-14	0.9	0.796	1.0	7.5E-13	0.8	0.642	1.0	2.7E-06	0.9	0.987	1.0
rs4077515	9:139266496	rs4075760 (R2=0.9959)	1.5E-25	1.2	0.297	1.1	4.4E-20	1.2	0.570	1.1	8.1E-13	1.2	0.269	1.1
rs12722515	10:6081230	rs12722515	0.001	0.9	0.059	0.8	2.6E-04	0.9	0.198	0.8	0.043	0.9	0.128	0.8
rs2050392	10:30691503	rs2050392	6.3E-05	1.1	0.510	1.1	3.1E-05	1.1	0.311	1.1	0.030	1.0	0.971	1.0
rs34779708	10:35466185	rs34779708	3.2E-11	0.9	0.051	0.9	2.0E-09	0.9	0.059	0.8	8.6E-07	0.9	0.447	0.9
rs2153283	10:59972299	rs2153283	4.8E-05	0.9	0.686	1.0	1.2E-06	0.9	0.355	0.9	0.036	0.9	0.602	1.1
rs10761659	10:64445564	rs10761659	4.1E-21	0.9	0.233	0.9	3.4E-19	0.8	0.403	0.9	2.2E-07	0.9	0.230	0.9
rs2688608	10:75658349	rs2688608	0.001	1.1	0.749	1.0	6.5E-06	1.1	0.404	0.9	0.445	1.0	0.653	1.1
rs1250566	10:81046453	rs1250566	3.0E-09	0.9	0.001	0.7	1.6E-10	0.8	0.028	0.8	0.003	0.9	0.007	0.7
rs7097656	10:82250831	rs7922621 (R2=1.0)	6.9E-06	0.9	0.858	1.0	1.3E-05	0.9	0.816	1.0	0.014	0.9	0.990	1.0
rs11187157	10:94502244	rs7924271 (R2=0.1564)	1.9E-05	0.9	0.011	1.2	0.104	1.0	0.011	1.3	0.001	0.9	0.145	1.2
rs10748781	10:101283330	rs10786557 (R2=0.7811)	4.3E-24	0.8	0.061	1.2	3.7E-20	0.8	0.058	1.2	1.9E-12	0.9	0.373	1.1
rs2274351	10:104264107	rs12766112 (R2=1.0)	2.2E-06	1.1	4.0E-04	0.7	0.003	1.1	0.094	0.8	3.1E-06	1.1	1.6E-04	0.6
rs907611	11:1874072	rs907611	8.3E-06	1.1	0.061	1.2	0.085	1.0	0.495	1.1	8.9E-07	1.1	0.007	1.4
rs11229555	11:58408687	rs11229555	9.7E-05	0.9	0.448	0.9	0.163	1.0	0.053	0.8	8.5E-05	0.9	0.255	1.1
rs11230563	11:60776209	rs2074227 (R2=0.9828)	2.8E-06	0.9	0.800	1.0	9.4E-05	0.9	0.855	1.0	0.002	0.9	0.841	1.0
rs1535	11:61597972	rs1535	1.0E-04	0.9	0.395	0.9	1.8E-06	0.9	0.614	0.9	0.078	1.0	0.438	0.9
rs559928	11:64150370	rs559928	3.1E-05	0.9	0.887	1.0	4.2E-06	0.9	0.317	1.1	0.056	0.9	0.425	0.9
rs732072	11:65427568	rs732072	0.014	0.9	0.378	1.1	0.024	0.9	0.543	1.1	0.068	0.9	0.465	1.2

rs11236797	11:76299649	rs11236797	4.8E-20	1.2	0.512	1.1	4.9E-15	1.2	0.778	1.0	1.0E-10	1.1	0.439	1.1
rs4397880	11:87083443	rs4397880	0.111	1.0	0.128	1.1	0.111	1.0	0.459	1.1	0.135	1.0	0.054	1.3
rs483905	11:96023427	rs483905	1.7E-06	1.1	0.406	0.9	0.279	1.0	0.519	0.9	1.6E-08	1.1	0.396	0.9
rs661054	11:114430410	rs661946 (R2=1.0)	7.4E-06	1.1	0.189	0.9	0.123	1.0	0.223	0.9	7.0E-09	1.1	0.251	0.9
rs3922	11:118765600	rs3922	0.052	1.0	0.771	1.0	0.075	1.0	0.532	1.1	0.298	1.0	0.778	1.0
rs7954567	12:6491125	rs7954567	0.001	1.1	0.630	1.0	7.0E-05	1.1	0.511	0.9	0.322	1.0	0.981	1.0
rs11054935	12:12648843	rs11054935	0.002	0.9	0.250	0.9	0.288	1.0	0.426	0.9	1.2E-04	0.9	0.262	0.9
rs1388585	12:40531691	rs1388585	1.5E-13	0.7	0.126	0.6	1.8E-12	0.6	0.027	0.5	1.0E-05	0.7	0.846	1.1
rs11168249	12:48208368	rs11168249	2.1E-05	0.9	0.223	1.1	0.075	1.0	0.193	1.1	6.7E-05	0.9	0.419	1.1
rs12318183	12:68503836	rs12318183	9.1E-15	1.1	0.057	1.2	0.007	1.1	0.060	1.2	4.2E-18	1.2	0.264	1.1
rs3184504	12:111884608	rs7310615 (R2=0.996)	2.0E-05	1.1	0.374	1.1	0.002	1.1	0.115	1.2	0.001	1.1	0.690	1.0
rs11064881	12:120146925	rs11064881	0.006	1.1	0.702	1.1	0.004	1.1	0.782	1.1	0.028	1.1	0.574	1.1
rs12585310	13:27528347	rs12585310	2.7E-04	1.1	0.986	1.0	0.498	1.0	0.645	1.1	1.2E-05	1.1	0.681	1.0
rs941823	13:41013977	rs941823	1.4E-06	0.9	0.881	1.0	0.026	0.9	0.741	1.0	1.6E-06	0.9	0.993	1.0
rs61959448	13:42910319	rs61959448	0.001	1.1	0.969	1.0	9.0E-06	1.1	0.821	1.0	0.312	1.0	0.632	0.9
rs6561151	13:44484706	rs9316058 (R2=1.0)	1.5E-04	1.1	0.643	0.9	1.7E-08	1.2	0.828	1.0	0.396	1.0	0.666	0.9
rs9557207	13:100036418	rs9557207	8.6E-06	1.1	0.654	1.0	2.5E-05	1.1	0.793	1.0	0.008	1.1	0.768	1.0
rs10142466	14:69271784	rs4902651 (R2=0.992)	2.7E-07	1.1	0.736	1.0	6.3E-08	1.1	0.997	1.0	0.013	1.1	0.617	0.9
rs1569328	14:75741751	rs1569328	0.003	0.9	0.398	0.9	0.001	0.9	0.596	0.9	0.052	0.9	0.348	0.8
rs55808324	14:88444752	rs55808324	5.7E-08	1.2	0.062	1.3	4.2E-07	1.2	0.054	1.4	9.9E-05	1.1	0.377	1.2
rs72727394	15:38847022	rs72727394	0.095	1.0	0.864	1.0	5.8E-06	1.1	0.606	1.1	0.277	1.0	0.548	0.9
rs2777491	15:41735710	rs2777491	0.002	1.1	0.590	1.0	0.833	1.0	0.426	0.9	9.5E-05	1.1	0.782	1.0
rs17293632	15:67442596	rs17293632	5.7E-14	1.2	0.296	1.1	1.1E-12	1.2	0.942	1.0	1.7E-05	1.1	0.129	1.2
rs7165170	15:91181489	rs7165170	0.003	1.1	0.493	1.1	0.002	1.1	0.846	1.0	0.033	1.1	0.321	1.1
rs11641184	16:11704651	rs11641184	2.6E-04	1.1	0.057	1.2	0.002	1.1	0.039	1.2	0.004	1.1	0.361	1.1
rs7404095	16:23864590	rs7404095	5.6E-06	0.9	0.620	1.0	0.005	0.9	0.917	1.0	1.5E-05	0.9	0.272	1.1
rs62037363	16:28865042	rs62037363	1.3E-09	0.9	0.550	1.0	1.5E-09	0.9	0.391	0.9	0.001	0.9	0.924	1.0
rs11150589	16:30482494	rs12598978 (R2=1.0)	3.0E-04	1.1	0.051	1.2	0.034	1.1	0.343	1.1	3.0E-04	1.1	0.022	1.3
rs12149608	16:68586504	rs12149608	4.5E-04	1.1	0.802	1.0	0.046	1.1	0.887	1.0	7.8E-05	1.2	0.884	1.0
rs11641016	16:86014881	rs11641016	5.1E-05	1.1	0.672	1.1	3.1E-04	1.1	0.156	1.2	0.030	1.1	0.337	0.9
rs2945412	17:25843643	rs2945412	0.433	1.0	0.302	1.1	1.6E-04	1.1	0.589	1.1	0.049	1.0	0.348	1.1
rs9889296	17:32570547	rs9889296	3.2E-08	0.9	0.001	0.7	1.2E-10	0.8	0.006	0.7	0.020	0.9	0.026	0.7
rs4795397	17:38023745	rs4795397	1.4E-17	0.9	0.257	1.1	2.6E-09	0.9	0.845	1.0	1.4E-11	0.9	0.086	1.2
rs744166	17:40514201	rs744166	2.2E-12	1.1	0.680	1.0	2.9E-08	1.1	0.757	1.0	2.0E-06	1.1	0.751	1.0
rs3853824	17:54880993	rs3853824	0.004	1.0	0.873	1.0	0.032	0.9	0.947	1.0	0.016	0.9	0.911	1.0
rs1292053	17:57963537	rs1292053	9.6E-05	0.9	0.554	1.0	8.5E-06	0.9	0.166	0.9	0.089	1.0	0.592	1.1
rs17780256	17:70642923	rs17780256	3.0E-06	1.1	0.033	1.2	0.002	1.1	0.050	1.3	1.6E-04	1.1	0.201	1.2
rs17736589	17:76737118	rs17736589	1.8E-04	0.9	0.850	1.0	0.099	1.0	0.999	1.0	7.9E-05	0.9	0.795	1.0
rs2847278	18:12778715	rs2847278	3.6E-11	0.9	0.170	0.9	4.8E-13	0.8	0.400	0.9	2.8E-04	0.9	0.161	0.8
rs7240004	18:46395022	rs7240004	7.0E-06	1.1	0.138	1.1	0.002	1.1	0.312	1.1	4.2E-05	1.1	0.167	1.2
rs1517037	18:56878274	rs1517037	0.008	1.1	0.398	1.1	3.6E-04	1.1	0.135	1.2	0.191	1.0	0.881	1.0
rs17207042	18:67537351	rs17207042	0.004	1.1	0.696	1.0	0.022	1.1	0.088	1.2	0.144	1.0	0.139	0.8
rs7236492	18:77220616	rs7236492	2.2E-04	0.9	0.669	1.1	0.028	0.9	0.646	1.1	0.003	0.9	0.732	1.1
rs2024092	19:1124031	rs2024092	2.7E-11	1.1	0.548	1.1	2.3E-11	1.2	0.364	1.1	2.8E-04	1.1	0.902	1.0
rs35164067	19:10525181	rs35164067	2.9E-07	0.9	0.240	0.9	1.7E-08	0.8	0.177	0.8	0.037	0.9	0.807	1.0
rs17694108	19:33731551	rs17694108	1.9E-07	1.1	0.366	1.1	2.3E-05	1.1	0.567	1.1	4.7E-06	1.1	0.327	1.1

rs4802307	19:46849806	rs4802307	0.005	0.9	0.931	1.0	0.001	0.9	0.906	1.0	0.498	1.0	0.968	1.0
rs11083840	19:47119910	rs11083840	0.001	0.9	0.863	1.0	0.275	1.0	0.378	1.1	4.4E-04	0.9	0.559	0.9
rs516246	19:49206172	rs516246	8.2E-05	1.1	0.316	1.1	1.2E-08	1.1	0.177	1.1	0.364	1.0	0.905	1.0
rs17771967	19:55380214	rs17771967	0.022	1.0	0.737	1.0	0.856	1.0	0.452	0.9	1.7E-04	0.9	0.277	1.1
rs4243971	20:30849517	rs4243971	2.8E-04	0.9	0.319	0.9	0.005	0.9	0.729	1.0	0.023	1.0	0.156	0.9
rs6058869	20:31348750	rs6058869	0.001	1.1	0.962	1.0	0.133	1.0	0.330	1.1	0.002	1.1	0.419	0.9
rs6088765	20:33799280	rs6088765	0.014	1.0	0.515	0.9	0.865	1.0	0.394	0.9	4.1E-04	0.9	0.811	1.0
rs4812833	20:43068996	rs4812835 (R2=0.8394)	0.001	1.1	0.828	1.0	0.373	1.0	0.903	1.0	1.1E-07	1.1	0.475	0.9
rs6074022	20:44740196	rs6032664 (R2=1.0)	1.4E-04	0.9	0.340	1.1	0.001	0.9	0.446	1.1	0.023	0.9	0.429	1.1
rs913678	20:48955424	rs913678	7.5E-06	1.1	0.933	1.0	0.010	1.1	0.976	1.0	1.3E-04	1.1	0.804	1.0
rs259964	20:57824309	rs259958 (R2=0.996)	5.7E-06	1.1	0.291	1.1	0.001	1.1	0.697	1.0	0.001	1.1	0.185	1.2
rs6062496	20:62329099	rs6062496	5.5E-20	1.2	0.056	1.2	7.2E-09	1.2	0.040	1.2	1.5E-12	1.2	0.529	1.1
rs1297258	21:16806709	rs1297258	1.4E-11	0.9	0.003	0.8	3.2E-10	0.9	0.014	0.8	1.6E-05	0.9	0.054	0.8
rs2284553	21:34776695	21:34776695:A:G	0.001	0.9	0.131	0.9	1.4E-06	0.9	0.009	0.8	0.337	1.0	0.727	1.0
rs2836883	21:40466744	rs2836883	1.7E-22	0.8	0.014	0.8	2.7E-06	0.9	0.094	0.8	7.6E-21	0.8	0.036	0.8
rs8127691	21:45614860	rs8127691	2.9E-17	1.2	0.098	1.1	1.6E-13	1.2	0.203	1.1	4.0E-10	1.1	0.135	1.2
rs2266961	22:21928597	rs2266961	5.4E-10	0.9	0.199	0.9	1.2E-08	0.9	0.538	0.9	1.2E-04	0.9	0.122	0.8
rs1003342	22:30570022	rs1003342	1.7E-08	1.1	0.073	1.2	2.8E-07	1.1	0.473	1.1	0.001	1.1	0.010	1.3
rs2143178	22:39660829	rs2143178	2.2E-12	1.2	4.6E-04	1.5	4.9E-10	1.2	0.038	1.3	8.2E-06	1.1	0.001	1.7
rs727563	22:41867377	rs727563	0.141	1.0	0.469	0.9	0.035	0.9	0.331	0.9	0.512	1.0	0.873	1.0

IBD, results from the analysis of all IBD patients; CD, results from the analysis of only Crohn`s Disease patients; UC, results from the analysis of only Ulcerative Colitis patients. IIBDGC, results from Internation IBD genetics consortium; BasqueIBD, results from the present work. P, p-value of the effect allele; OR, odds-ratio of the effect allele.

Supplementary Table S3: Genome-Wide Association Studies of IBD, its subtypes and analysed location and extension for selected genes. Only pruned SNPs (as representative of their linkage-disequilibrium block) are showed.

	**IBD**				**CD**				**UC**				**Ileal CD**		**Ileocolonic CD**		**Left UC**		**Pancolitis UC**	
	**IIBDGC**		**BasqueIBD**		**IIBDGC**		**BasqueIBD**		**IIBDGC**		**BasqueIBD**		**BasqueIBD**		**BasqueIBD**		**BasqueIBD**		**BasqueIBD**	
	**P**	**OR**	**P**	**OR**	**P**	**OR**	**P**	**OR**	**P**	**OR**	**P**	**OR**	**P**	**OR**	**P**	**OR**	**P**	**OR**	**P**	**OR**
**IL23R**																				
rs7532161	0.001	0.9	0.093	0.9	3.79E-08	0.9	0.172	0.9	0.660	1.0	0.151	0.9	0.689	0.9	0.020	0.7	0.663	0.9	0.225	0.8
rs12565567	0.546	1.0	0.345	1.2	0.295	1.1	0.109	1.4	0.071	0.9	0.780	0.9	0.324	1.3	0.081	1.7	0.750	1.1	0.524	0.7
rs113811273	0.002	1.3	0.386	1.3	4.85E-04	1.4	0.931	1.0	0.387	1.1	0.119	1.7	0.766	0.9	0.552	0.7	0.559	1.3	0.086	2.1
rs76231630	NA	NA	0.020	0.6	NA	NA	0.043	0.5	NA	NA	0.190	0.6	0.255	0.6	0.046	0.3	0.519	0.8	0.565	0.8
rs61780314	0.666	1.0	0.338	1.2	0.534	1.0	0.044	1.6	0.194	1.1	0.427	0.8	0.272	1.4	0.024	2.0	0.955	1.0	0.202	0.4
rs116360994	0.199	1.1	0.496	0.8	0.338	1.1	0.372	0.7	0.207	1.1	0.917	1.0	0.599	0.8	0.484	0.6	0.556	0.7	0.612	0.7
rs112874012	2.60E-08	0.8	0.563	1.1	0.001	0.8	0.983	1.0	3.17E-04	0.8	0.294	1.3	0.599	1.2	0.988	1.0	0.177	1.6	0.445	1.4
rs114316017	8.11E-07	1.3	0.717	0.9	1.14E-07	1.4	0.449	1.2	0.009	1.2	0.191	0.6	0.102	1.7	0.995	1.0	0.389	0.6	0.167	0.2
rs185138068	NA	NA	0.080	0.6	NA	NA	0.064	0.5	NA	NA	0.541	0.8	0.427	0.7	0.080	0.3	0.716	0.8	0.853	1.1
rs11209023	2.03E-31	1.2	0.010	0.8	1.89E-31	1.3	0.005	0.7	2.08E-10	1.1	0.288	0.9	0.021	0.7	0.028	0.7	0.539	0.9	0.635	0.9
rs78134424	0.295	1.1	0.286	0.7	0.477	1.1	0.242	0.6	0.265	1.1	0.663	0.8	0.572	0.7	0.244	0.4	0.266	0.4	0.643	0.7
rs12070470	0.599	1.0	0.263	0.9	0.749	1.0	0.405	0.9	0.830	1.0	0.456	0.9	0.784	0.9	0.602	0.9	0.878	1.0	0.414	0.8
rs7542081	0.162	1.0	0.738	1.0	0.285	1.0	0.876	1.0	0.127	1.0	0.454	0.9	0.678	1.1	0.610	1.1	0.731	0.9	0.857	1.0
rs114871562	1.76E-06	1.2	0.799	0.9	1.90E-07	1.4	0.477	1.2	0.015	1.2	0.304	0.7	0.104	1.7	0.938	1.0	0.522	0.7	0.263	0.4
rs10489628	4.12E-05	0.9	0.556	1.0	4.02E-06	0.9	0.249	0.9	0.125	1.0	0.566	1.1	0.258	0.8	0.283	0.9	0.591	1.1	0.573	1.1
rs12751814	8.80E-05	1.1	0.468	0.9	1.54E-05	1.1	0.221	0.9	0.081	1.0	0.835	1.0	0.407	0.9	0.296	0.9	0.867	1.0	0.731	1.1
rs115917484	NA	NA	0.472	0.8	NA	NA	0.257	0.7	NA	NA	0.828	1.1	0.705	0.8	0.131	0.3	0.638	1.2	0.581	1.3
rs72676094	0.001	0.8	0.924	1.0	3.38E-05	0.8	0.995	1.0	0.383	0.9	0.957	1.0	0.805	1.1	0.330	0.6	0.959	1.0	0.996	1.0
rs113860798	2.77E-05	1.3	0.343	0.7	0.056	1.2	0.485	0.8	0.001	1.2	0.440	0.7	0.698	0.8	0.535	0.7	0.813	1.1	0.327	0.4
rs79398380	NA	NA	0.561	1.2	NA	NA	0.159	1.7	NA	NA	0.489	0.6	0.263	1.8	0.198	1.9	0.870	0.9	0.598	0.6
rs10889675	0.255	1.0	0.410	1.1	0.346	1.0	0.315	1.2	0.367	1.0	0.768	1.1	0.561	1.1	0.390	1.2	0.545	1.1	0.640	1.1
rs78691454	NA	NA	0.989	1.0	NA	NA	0.855	0.9	NA	NA	0.804	1.1	0.812	0.9	0.971	1.0	0.921	1.1	0.425	1.7
**ATG16L1**																				
rs78446401	0.012	0.9	0.777	1.0	2.64E-04	0.8	0.614	0.9	0.431	1.0	0.749	1.1	0.780	0.9	0.407	0.8	0.840	0.9	0.980	1.0
rs73110098	0.006	0.9	0.360	0.9	1.04E-08	0.7	0.606	0.9	0.715	1.0	0.243	0.8	0.363	0.8	0.551	0.8	0.259	0.7	0.166	0.5
rs78778324	0.007	1.1	0.546	0.9	2.43E-04	1.2	0.602	0.9	0.324	1.0	0.820	0.9	0.812	0.9	0.562	0.8	0.439	0.8	0.863	0.9
rs6753686	0.002	0.9	0.929	1.0	6.50E-07	0.8	0.952	1.0	0.654	1.0	0.825	1.0	0.792	0.9	0.613	1.1	0.305	1.3	0.441	0.8
rs4663420	0.007	1.1	0.375	0.9	4.94E-09	1.4	0.613	0.9	0.646	1.0	0.256	0.8	0.364	0.8	0.559	0.8	0.261	0.7	0.169	0.5
**IRGM**																				
rs17111376	7.34E-11	0.8	0.865	0.9	2.92E-17	0.7	0.464	1.1	0.002	0.9	0.210	0.8	0.473	1.1	0.699	1.1	0.174	0.7	0.354	0.8
**TNFSF15**																				
rs78979172	NA	NA	0.632	0.9	NA	NA	0.617	0.8	NA	NA	0.737	0.9	0.325	0.5	0.825	0.9	0.776	1.2	NA	NA
**LRRK2**																				
rs17465737	NA	NA	0.627	0.9	NA	NA	0.392	0.7	NA	NA	0.881	1.1	0.327	0.6	0.685	0.8	0.964	1.0	0.328	1.5
rs1491942	2.38E-05	0.9	0.031	1.2	4.16E-05	0.9	0.016	1.3	0.003	0.9	0.461	1.1	0.077	1.3	0.110	1.3	0.774	1.1	0.881	1.0
rs17465751	NA	NA	0.117	0.7	NA	NA	0.062	0.5	NA	NA	0.841	0.9	0.552	0.8	0.063	0.3	0.349	1.4	0.318	0.5
rs150540643	4.19E-13	1.5	0.222	1.5	4.82E-14	1.6	0.114	1.8	2.77E-04	1.3	0.866	1.1	0.490	1.5	0.081	2.3	0.439	1.5	0.746	0.7
rs148217035	0.662	1.0	0.649	0.9	0.338	1.1	0.204	0.6	0.789	1.0	0.555	1.3	0.348	0.6	0.612	0.7	0.288	1.6	0.781	0.8

rs2131088	0.862	1.0	0.019	1.5	0.979	1.0	0.044	1.5	0.940	1.0	0.067	1.5	0.011	1.9	0.778	1.1	0.041	1.8	0.698	1.2
rs11564187	3.31E-14	0.7	0.139	1.6	2.60E-15	0.6	0.107	1.8	2.34E-04	0.8	0.988	1.0	0.335	1.6	0.111	2.1	0.514	1.4	0.682	0.7
rs1388599	0.241	1.0	0.248	0.9	0.014	1.1	0.246	0.8	0.589	1.0	0.544	0.9	0.169	0.7	0.791	0.9	0.887	1.0	0.768	0.9
rs73106354	NA	NA	0.319	0.7	NA	NA	0.224	0.6	0.107	1.1	0.934	1.0	0.117	0.3	0.629	0.8	0.876	1.1	0.527	0.6
rs17443407	0.600	1.0	0.053	0.7	0.052	0.9	0.133	0.7	0.292	1.0	0.187	0.7	0.490	0.8	0.209	0.6	0.385	0.7	0.301	0.6
rs1491939	0.741	1.0	0.024	1.5	0.464	1.0	0.082	1.4	0.771	1.0	0.032	1.6	0.033	1.8	0.796	1.1	0.015	2.0	0.673	1.2
rs142152465	0.143	1.1	0.591	1.2	0.315	1.1	0.607	1.3	0.202	1.1	0.734	1.2	0.652	0.7	0.653	1.3	0.470	0.5	0.705	1.3
rs2708447	NA	NA	0.956	1.0	NA	NA	0.439	1.4	NA	NA	0.454	0.6	0.304	1.7	0.751	1.2	NA	NA	0.816	1.2
rs118076169	NA	NA	0.091	1.9	NA	NA	0.072	2.1	NA	NA	0.515	1.4	0.677	1.3	0.026	2.8	0.450	1.6	0.925	0.9
rs11564152	1.71E-13	0.7	0.071	1.8	1.48E-15	0.6	0.038	2.1	0.001	0.8	0.988	1.0	0.073	2.2	0.111	2.1	0.514	1.4	0.682	0.7
rs11175769	0.049	1.0	0.710	1.0	0.167	1.0	0.226	0.9	0.128	1.0	0.442	1.1	0.262	0.9	0.101	0.8	0.720	0.9	0.141	1.3
rs11564113	0.915	1.0	0.397	0.7	0.891	1.0	0.370	0.7	0.993	1.0	0.763	0.9	0.461	0.6	0.337	0.5	0.374	0.5	0.701	1.3
rs146794882	0.173	0.9	0.501	0.9	0.158	0.9	0.846	0.9	0.460	1.0	0.457	0.8	0.392	0.7	0.266	1.5	0.481	0.7	0.979	1.0
rs147136292	0.813	1.0	0.400	0.7	0.112	1.2	0.385	0.7	0.821	1.0	0.650	0.8	0.532	0.7	0.796	0.9	0.711	1.2	0.477	0.5
rs17443607	0.935	1.0	0.036	1.5	0.636	1.0	0.025	1.7	0.496	1.0	0.356	1.3	0.188	1.6	0.030	2.0	0.241	1.5	0.492	0.7
rs186675266	0.020	1.1	0.652	1.1	0.010	1.2	0.587	1.2	0.350	1.1	0.719	1.1	0.241	1.6	0.914	0.9	0.623	1.2	0.847	0.9
rs144463374	0.510	1.0	0.348	0.8	0.568	1.0	0.482	0.8	0.950	1.0	0.504	0.8	0.271	0.5	0.935	1.0	0.568	1.3	0.260	0.3
rs17443656	7.33E-14	1.5	0.071	1.8	1.91E-15	1.6	0.038	2.1	0.001	1.3	0.988	1.0	0.073	2.2	0.111	2.1	0.514	1.4	0.682	0.7
rs10506150	0.016	1.1	0.928	1.0	0.074	1.1	0.996	1.0	0.032	1.1	0.960	1.0	0.531	0.9	0.876	1.0	0.633	0.9	0.382	1.3
rs17443670	0.650	1.0	0.835	1.1	0.188	0.9	0.616	0.8	0.747	1.0	0.408	1.3	0.962	1.0	0.509	0.7	0.173	1.7	0.798	0.9
rs7971935	0.205	1.0	0.313	0.9	0.019	0.9	0.188	0.8	0.763	1.0	0.855	1.0	0.309	0.8	0.767	0.9	0.473	1.2	0.755	0.9
rs11564182	0.711	1.0	0.197	0.7	0.194	0.9	0.072	0.4	0.635	1.0	0.979	1.0	0.247	0.5	0.364	0.6	0.789	1.1	0.847	1.1
rs149331112	0.990	1.0	0.354	0.7	0.866	1.0	0.248	0.6	0.966	1.0	0.884	0.9	0.362	0.6	0.272	0.4	0.325	0.5	0.431	1.5
rs11175847	0.066	1.0	0.794	1.0	0.429	1.0	0.320	0.9	0.076	1.0	0.490	1.1	0.338	0.9	0.126	0.8	0.458	0.9	0.118	1.3
rs11175852	0.042	0.9	0.057	0.7	2.58E-04	0.8	0.101	0.7	0.608	1.0	0.302	0.8	0.304	0.7	0.174	0.7	0.527	0.8	0.468	0.8
rs11564150	0.208	1.0	0.314	0.9	0.018	1.1	0.199	0.8	0.746	1.0	0.813	1.0	0.224	0.7	0.806	0.9	0.506	1.2	0.740	0.9
rs138250350	0.776	1.0	0.699	1.1	0.348	0.9	0.430	1.3	0.570	1.0	0.788	0.9	0.735	0.8	0.350	1.6	0.541	1.4	0.507	0.5
rs76434788	0.709	1.0	0.927	1.0	0.261	1.1	0.627	0.9	0.802	1.0	0.527	1.2	0.918	1.0	0.661	0.8	0.234	1.6	0.714	0.8
rs73102738	NA	NA	0.310	0.7	NA	NA	0.110	0.5	NA	NA	0.700	1.2	0.184	0.4	0.298	0.5	0.957	1.0	0.714	0.8
rs4768226	8.95E-14	1.5	0.056	1.8	1.80E-15	1.6	0.029	2.2	0.001	1.3	0.931	1.0	0.064	2.3	0.090	2.2	0.478	1.5	0.716	0.7
rs10878336	0.042	1.1	0.087	0.8	0.001	1.2	0.156	0.7	0.647	1.0	0.352	0.8	0.526	0.8	0.165	0.6	0.663	0.9	0.444	0.8
rs11564269	0.638	1.0	0.626	0.9	0.378	1.1	0.513	0.8	0.782	1.0	0.988	1.0	0.427	0.6	0.623	0.7	0.677	0.8	0.735	1.2
rs17484286	0.129	1.0	0.081	1.3	0.047	0.9	0.098	1.3	0.758	1.0	0.260	1.3	0.143	1.4	0.132	1.4	0.335	1.3	0.921	1.0
rs117997005	0.896	1.0	0.266	1.4	0.991	1.0	0.224	1.5	0.405	0.9	0.956	1.0	0.294	1.6	0.340	1.5	0.948	1.0	0.653	0.7
rs35507033	NA	NA	0.272	0.6	NA	NA	0.577	0.8	NA	NA	0.265	0.5	0.417	0.5	0.491	0.6	0.298	0.3	0.852	1.2
rs7132073	0.140	1.0	0.415	0.9	0.006	1.1	0.285	0.8	0.838	1.0	0.861	1.0	0.259	0.8	0.995	1.0	0.635	1.1	0.999	1.0
rs78774467	0.022	1.1	0.751	1.1	0.009	1.2	0.900	1.0	0.447	1.0	0.578	1.2	0.531	1.3	0.844	0.9	0.717	1.2	0.747	1.2
rs17466360	0.175	1.1	0.486	0.8	0.148	1.1	0.597	0.8	0.450	1.0	0.709	0.9	0.578	0.8	0.860	1.1	0.440	0.7	0.498	1.3
rs35303786	0.926	1.0	0.378	1.4	0.130	0.9	0.157	1.8	0.444	1.1	0.828	0.9	0.310	1.8	0.535	1.5	0.832	1.2	NA	NA
rs10878371	0.615	1.0	0.152	1.1	0.733	1.0	0.138	1.2	0.770	1.0	0.554	1.1	0.326	1.1	0.035	1.3	0.238	1.2	0.638	0.9
rs17444068	0.984	1.0	0.536	0.8	0.703	1.0	0.339	0.7	0.981	1.0	0.895	1.1	0.583	0.7	0.250	0.4	0.605	0.7	0.457	1.5
rs3747891	0.052	1.1	0.059	0.7	0.001	1.2	0.123	0.7	0.585	1.0	0.271	0.8	0.297	0.7	0.160	0.6	0.496	0.8	0.436	0.8
rs17444075	NA	NA	0.248	0.6	0.770	1.0	0.433	0.7	NA	NA	0.342	0.6	0.556	0.7	0.567	0.7	0.729	0.8	NA	NA
rs2404832	0.263	1.0	0.264	0.9	0.805	1.0	0.559	0.9	0.021	1.0	0.190	0.9	0.761	1.0	0.510	0.9	0.242	0.8	0.568	0.9
rs149596967	2.70E-13	0.7	0.061	1.8	5.31E-16	0.6	0.041	2.1	0.005	0.8	0.866	1.1	0.053	2.4	0.192	1.9	0.443	1.5	0.741	0.7
rs4767970	6.47E-07	0.9	1.84E-04	1.5	3.51E-08	0.8	3.34E-04	1.6	0.057	0.9	0.026	1.4	1.52E-04	2.0	0.043	1.5	0.017	1.6	0.925	1.0

rs17491536	0.308	1.0	0.517	0.9	0.245	1.1	0.686	0.9	0.720	1.0	0.471	0.9	0.813	1.1	0.340	0.8	0.174	0.6	0.732	1.1
rs10459264	0.057	0.9	0.044	0.7	1.07E-04	0.8	0.138	0.7	0.490	1.0	0.174	0.7	0.281	0.7	0.334	0.8	0.225	0.7	0.565	0.8
rs10459265	0.021	1.0	0.199	0.9	0.013	0.9	0.257	0.9	0.411	1.0	0.517	0.9	0.284	0.9	0.059	0.8	0.181	0.8	0.748	1.1
rs148319899	2.19E-14	0.7	0.061	1.8	6.93E-17	0.6	0.041	2.1	0.002	0.8	0.866	1.1	0.053	2.4	0.192	1.9	0.443	1.5	0.741	0.7
rs58911468	0.114	1.0	0.325	1.1	0.032	0.9	0.076	1.3	0.206	1.0	0.675	0.9	0.578	1.1	0.284	1.2	0.397	0.8	0.736	0.9
rs77668961	0.551	1.0	0.386	0.9	0.493	1.0	0.748	0.9	0.809	1.0	0.231	0.8	0.681	1.1	0.425	0.8	0.050	0.5	0.645	1.2
rs117224507	NA	NA	0.674	0.9	NA	NA	0.932	1.0	NA	NA	0.582	0.7	0.490	0.6	0.551	0.6	0.695	0.8	0.737	1.3
rs11564146	1.44E-14	0.7	0.061	1.8	3.32E-16	0.6	0.022	2.2	0.001	0.8	0.869	0.9	0.022	2.5	0.332	1.6	0.621	1.3	0.612	0.6
rs17466570	NA	NA	0.961	1.0	NA	NA	0.826	1.1	NA	NA	0.871	1.0	0.999	1.0	0.953	1.0	0.372	0.7	0.118	1.7
**NOD2**																				
rs75391704	NA	NA	0.937	1.0	NA	NA	0.736	0.9	NA	NA	0.802	1.1	0.314	0.7	0.865	0.9	0.829	0.9	0.175	1.6
16:50728498:G:C	NA	NA	0.891	1.0	NA	NA	0.673	1.2	NA	NA	0.810	0.9	0.530	0.7	0.912	1.1	0.422	0.6	0.704	1.3
rs5743263	0.381	1.0	0.217	0.8	0.556	1.0	0.067	0.7	0.473	1.0	0.859	1.0	0.211	0.7	0.409	0.8	0.706	0.9	0.661	0.8
rs5743292	NA	NA	0.003	2.4	NA	NA	4.82E-05	3.5	NA	NA	0.931	1.0	0.001	3.8	3.29E-04	3.9	0.559	0.6	0.364	1.8
rs72796367	NA	NA	0.138	0.5	NA	NA	0.136	0.4	NA	NA	0.566	0.7	0.788	0.8	NA	NA	0.822	1.2	0.506	0.5

IBD, results from the analysis of all IBD patients; CD, results from the analysis of only Crohn`s Disease patients; UC, results from the analysis of only Ulcerative Colitis patients. IIBDGC, results from Internation IBD genetics consortium; BasqueIBD, results from the present work. P, p-value of the effect allele; OR, odds-ratio of the effect allele.
